# The Use of Enhanced Analytical Pipelines for the Characterization of Poly(A) and Poly(A)-LNP Formulation Critical Quality Attributes

**DOI:** 10.1021/acs.molpharmaceut.5c00614

**Published:** 2025-10-30

**Authors:** Callum G. Davidson, Eleni Kapsali, Savvas Ioannou, Bojan Kopilovic, Muattaz Hussain, Yvonne Perrie, Zahra Rattray

**Affiliations:** † Strathclyde Institute of Pharmacy and Biomedical Sciences, 3527 University of Strathclyde, Glasgow G4 0RE, U.K.; ‡ School of Molecular Biosciences, University of Glasgow, Glasgow G11 6EW, U.K.; § School of Chemical, Materials and Biological Engineering, University of Sheffield, Sheffield S1 3JD, U.K.

**Keywords:** EAF4, drug substance, drug product, Poly(A), LNP

## Abstract

The number of nucleic acid therapeutics is set to grow within the pharmaceutical industry sector, deploying nanocarrier-based delivery systems as drug products. Poly­(A) is a widely used model sequence used in lipid nanoparticle (LNP) formulations for which there are no reported critical quality attributes (CQAs) such as molecular weight, chain length, and impurity profile. In this study, we analyze Poly­(A) from three different vendors to measure any existing differences in their CQAs. Poly­(A) from these brands was encapsulated in SM102 LNPs using microfluidics to produce three branded Poly­(A)-based LNPs. We utilized an orthogonal analytical pipeline approach for both Poly­(A) drug substance and LNP drug product CQA evaluation, which included a combination of dynamic light scattering and flow field flow fractionation multiplexed with inline UV, dynamic, and multiangle light-scattering detectors. Similar purity (260/280) values of >3 were obtained across all three brands of Poly­(A); however, distinct differences in molecular weight and chain length distributions were identified across Poly­(A) brands, with this study representing the first to apply EAF4 methodology for in-depth characterization of model RNA drug substances. Brand A produced a smaller and broader molecular weight distribution, followed by Brand B, and then Brand C produced the largest molecular weight species and the most uniform molecular weight distribution. On encapsulation in LNPs, differences seen in Poly­(A) CQAs did not translate to CQA differences in resultant LNPs. We show that a deeper understanding of drug substance CQAs and their subsequent impact on resultant overall drug product characteristics is needed on a case-by-case basis. We show correlations between analytical pipelines, with future work investigating the impact of RNA molecular weight in LNP formulations with different lipid compositions and using these correlations in AI or machine learning to further enhance our knowledge of the correlation between drug substance and resultant drug product CQAs.

## Graphical abstract

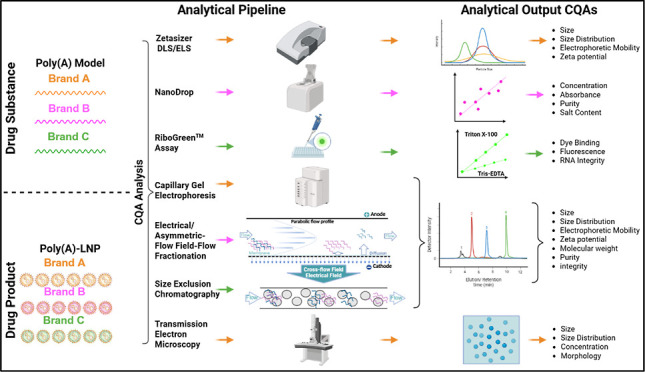

## 1 Introduction

Nucleic acid therapeutics have gained significant attention in the pharmaceutical and drug delivery fields^1^ owing to the global implementation of the COVID-19 mRNA mass vaccination programs. The successful clinical translation of ribonucleic acid-encapsulated lipid nanoparticle (RNA-LNP) drug delivery systems has played a pivotal role in this surge, with the market expected to grow to $48 billion by 2036.^2^ This momentum is further driven by the U.S. Food and Drug Administration (FDA) approval of RNA-LNP-based therapies, including Onpattro, Comirnaty, Spikevax,^3–5^ and, most recently, the Moderna respiratory syncytial virus vaccine mRESVIA in 2024.^6^

Drug delivery systems are engineered to protect encapsulated RNA cargo against degradation, ensuring safe and effective delivery to the target intracellular domains. Unlike conventional drugs, RNA drug substances bypass the need for an active protein-mediated therapeutic site by binding to endogenous cellular RNAs, halting or regulating disease pathways. This paradigm shift has led to the discovery of candidate RNA drug substances against a broad spectrum of novel therapeutic targets.^7–12^ However, due to their inherently large and hydrophilic nature, RNA therapeutics face unique translational challenges, requiring enhanced chemical modifications or advanced delivery systems. Chemical modifications to RNA-based drug substances can be introduced at the phosphate backbone, ribose sugar, or nucleobase.^8,13,14^ These modified RNAs can be encapsulated in a variety of delivery systems including antibodies, lipids, polymers, and nanoparticles,^9,11,12^ with lipid nanoparticles (LNPs) being prominent due to their formulation simplicity. RNA-LNPs are typically produced by microfluidic mixing of an organic lipid phase with an aqueous RNA phase, followed by purification into a neutral buffer prior to analysis of physicochemical critical quality attributes.

Polyadenylic acid (Poly­(A)) has emerged as a popular choice for use as a model RNA drug substance for LNP formulation prototyping, ranging from LNPs manufactured with cationic-LNPs^15,16^ to ionizable-LNPs^17–19^ and different structured ionizable-LNPs.^20–22^ In the absence of existing RNA and LNP drug substance/drug product reference materials, Poly­(A)-LNPs are widely used as a control formulation to assess the impact of manufacturing process parameters on formulation critical quality attributes (CQAs). Since the stability of RNA drug substance cargo is a major determinant of drug product performance, both preformulation and postformulation attributes of the RNA must be assessed. In particular, the source of the RNA, i.e., the RNA manufacturer, may influence these attributes, highlighting the need for a systematic comparison of drug substance quality and its effect on drug product formulation.

A range of analytical techniques are used to quantify RNA drug substance attributes, ranging from chromatographic to light scattering, electrophoretic, and mass spectrometry-based methods. Chromatographic approaches remain the gold standard, with advancements in separation modalities expanding the separation toolbox to include a larger repertoire of column stationary phases, including hydrophilic interaction liquid chromatography,^23–25^ ion exchange,^26,27^ hydrophobic ion-pairing chromatography,^28–30^ and aqueous-based separation techniques such as size exclusion chromatography.^31–33^ More recently, flow field-flow fractionation (FFF),^34–36^ coupled with light scattering-based detectors, has enabled high-resolution interrogation of nucleic acid and LNP size distribution profiles based on analyte molecular diffusion for size-based detection. Electrophoresis is another routine technique for electrical-based separation^37,38^ and detection of multiple molecular weight RNA species with agarose gel systems and specialist capillary electrophoretic instruments being adopted as analytical methods.^39,40^ Beyond advancements in separation technologies, detection methods multiplexed to separation-based approaches have seen routine adoption of triple quadrupole mass spectrometry and ion-mobility mass spectrometry for high-resolution detection of purity and conformational analysis, respectively.^41–44^

Despite the widespread use of Poly­(A) in LNP formulation studies, critical quality attributes of Poly­(A) remain under-characterized, with previous studies focusing on Poly­(A) in the context of therapeutic mRNA tails (>100 bases),^28,45,46^ and further impact on mRNA translation.^47–49^ Therefore, comprehensive comparative analyses of drug substance sequences from different manufacturers are currently lacking, highlighting the need for deeper investigation of these sequences as model nucleic acid drugs. In this study, we assess three commercially available Poly­(A) drug substances from three anonymized manufacturers (Brand A, Brand B, and Brand C) and evaluate the impact of their encapsulation in a model LNP formulation as three formulations referred to as A-LNP, B-LNP, and C-LNP.

This study aims to enhance knowledge of the model Poly­(A) critical quality attributes impact on LNP formulation attributes, ultimately seeking to produce better standardized controls for LNP bench-to-bedside translation. We demonstrate that the chain length distribution associated with different Poly­(A) brands varies, using a variety of preformulation and postformulation buffers. Our findings demonstrate that despite observable Poly­(A) molecular weight differences, SM102-LNPs exhibited no significant differences in variation of their associated drug product CQAs. However, further work is needed to evaluate the impact of Poly­(A) drug substance CQA variability in LNP drug products manufactured with differing lipid compositions and ratios.

## 2 Materials and Methods

### 2.1 Materials

Poly­(A) was purchased from three manufacturers (Merck, Roche, and Cytiva). DNA/RNA-free water, 10× phosphate-buffered saline (PBS) pH 7.4, sodium citrate dihydrate, and the Quant-it RiboGreen RNA Quantification Assay kit were acquired from ThermoFisher (Fisher Scientific, Leicestershire, UK), and 1 N hydrochloric acid was sourced from Alfa Aesar.

8-[(2-Hydroxyethyl)­[6-oxo-6-83 (undecyloxy)­hexyl]­amino]-octanoic acid and 1-octylnonyl ester (SM-102) were purchased from BroadPharm (San Diego, CA, USA). 1,2-Distearoyl-*sn*-glycero-3-phosphocholine (DSPC) and 1,2-dimyristoyl-rac-*glycero*-3-methoxypolyethylene glycol-2000 (DMG-PEG 2000) were purchased from Avanti Polar Lipids 81 (Alabaster, AL, USA). Cholesterol (CHOL) and Amicon-15 100 kDa MWCO regenerated cellulose spin columns were sourced from Sigma-Aldrich (Merck, Gillingham, UK).

### 2.2 Methods

Methods include the analysis of Poly­(A) within several buffer types, citrate pH 6 (50 mM), citrate pH 4 (50 mM) representing preformulation LNP buffers, and 1× PBS (pH 7.4), representing a neutral and biocompatible buffer for Poly­(A) drug substance use, which is also reflective of the Poly­(A) buffer combination post-LNP formulation purification.

#### 2.2.1 Determination of Sample Concentration

A NanoDrop was used to verify Poly­(A) sample concentration, accounting for lyophilized salt content within gravimetrically weighted samples. Analyte concentration was calculated using the Beer–Lambert Law (eq 1).

1 A=ε×c×l

where *A* is the absorbance, ε is the molar absorptivity constant, *c* is the concentration, and *l* is the path length, with an absorbance value equal to 1 representing 40 μg of single-stranded RNA. Calibration curves (0.5–2.0 mg/mL) Poly­(A) were prepared from a 10 mg/mL stock (DNA/RNA water) and diluted in either PBS or 50 mM citrate buffer (pH 4 and pH 6) to perform concentration calibrations. Samples representing each concentration were measured at 260 nm using NanoDrop to evaluate RNA dilution linearity and the NanoDrop limit of detection and limit of quantification.

#### 2.2.2 RiboGreen Assay

The integrity of branded Poly­(A) was measured using the Quant-it RiboGreen RNA Quantitation assay Kit (Thermo Fisher no. 4110) and using 0–1000 ng/mL calibration curves investigating the impact of Triton X-100 on Poly­(A) and fluorescent dye binding using TE buffer. As Triton X-100 is used to lyse Poly­(A)-LNPs and facilitate the release of encapsulated Poly­(A), its potential impact on RNA sample integrity was carefully assessed. In addition, the assay linearity, limit of detection (LOD), and limit of quantification (LOQ) were evaluated for each Poly­(A) drug substance to ensure assay robustness and reliability across all samples.

The fluorescence intensity signal of RiboGreen was measured on a GloMax Explorer GM3500 microplate reader (Promega, UK) at an excitation wavelength of 475 nm, with the emitted fluorescence measured at 500–550 nm. All fluorescence data were captured at ambient temperature (25 °C) using GloMax firmware version 4.29.0 and processed using GloMax Fluorescence software version 3.1.0.

#### 2.2.3 Dynamic Light Scattering

Particle size (*Z*-average) and polydispersity index (PDI) were measured by dynamic light scattering (DLS) using a Zetasizer Nano ZS system (Malvern Panalytical, Worcestershire, UK) equipped with a 633 nm Helium–Neon laser and a detection angle of 173° (noninvasive back scattering). Unless otherwise stated, all measurements were performed at 25 °C and at a 1:100 dilution from stock 10 mg/mL in DNA/RNA-free water to 0.1 mg/mL in the corresponding buffer. All measurements were performed in three independent replicate measurements consisting of at least two technical replicates.

The diffusion coefficient at different concentrations (0.5–6.25 mg/mL) was measured for branded Poly­(A) samples in PBS and 50 mM citrate buffer (pH 6 and pH 4) and to subsequently calculate the diffusion self-interaction parameter (*K*_D_), a measure of self-association (eq 2).^50^

2 $$D=D_{0}\left(1+K_{D}c+...\right)$$

where *D* is the diffusion coefficient, *D*_0_ is the maximum diffusion coefficient at infinite solute dilution, and *c* is the concentration of the solute in the solution.

#### 2.2.4 Electrical Asymmetric Flow Field Flow Fractionation (EAF4)

An AF2000 Asymmetric-Flow Field-Flow Fractionation (AF4) module (Postnova Analytics, Germany), hyphenated with multiple inline detectors including a multiangle light scattering (MALS-PN3621, Postnova Analytics), a UV detector (PN3242, 260 nm, Postnova Analytics), and a DLS Zetasizer Nano ZS system (Malvern Panalytical, Worcestershire, UK), was used to perform separation and inline analysis of Poly­(A). An electrical AF4 separation channel, with a channel spacer thickness of 500 μm and a 10 kDa molecular weight cutoff (MWCO) amphiphilic regenerated cellulose membrane, was used for Poly­(A) separation, with a 100 μL sample injection loop and an injection volume of 20 μL. Phosphate buffer (1.25 mM, pH 7.4) was used as the carrier liquid, and an injection flow rate of 0.2 mL/min, a cross-flow rate of 1.5 mL/min (linear power decay), and a detector flow rate of 0.5 mL/min were used as elution conditions. Neutral (0.0 mA) and positive (+1.0 mA) currents were applied to the EAF4 channel. Each Poly­(A) sample was injected in three technical replicates (0.25 mg/mL). Poly­(A) recovery (% Rec) was calculated as per an established protocol.^16^ Optimized method quality was verified against the International Organisation for Standardisation standard ISO/TS/21362:2021.^51^

The molecular weight and molar mass distributions of Branded Poly­(A)­s were determined using direct injection parameters (neutral current, 0.5 mL/min tip flow, 0.0 mL/min cross-flow for 25 min, 12.5 μg sample) into a hyphenated detector series. Direct injection traces were processed at a full-width half-maximum (FWHM) MALS-90° detector signal using 52–124° detector angles and random coil data fitting.

#### 2.2.5 Electrophoretic Light Scattering (ELS)

The ζ-potential surface charge was measured using ELS. Unless otherwise stated, all measurements were performed at 25 °C and at an equivalent dilution factor to the EAF4 channel in phosphate buffer (1.25 mM, pH 7.4). All ζ-potential measurements were performed using two independent replicates consisting of at least two technical measurements.

#### 2.2.6 Size Exclusion Chromatography (SEC)

Analytical SEC (aSEC) was performed on the AF2000 Asymmetric-Flow Field-Flow Fractionation (AF4) module (Postnova Analytics, Germany), hyphenated with multiple inline detectors (as per Section 2.2.4), using a TOSOH TSKgel UP-SW2000 column (300 × 4.6 mm, 2 μm) and 1× PBS pH 7.4 isocratic mobile phase with a corresponding flow rate of 0.2 mL/min and equivalent injection concentration to that in Section 2.2.4. The UV detector sensitivity was reduced to a +1.0 output.

#### 2.2.7 Capillary Gel Electrophoresis (CGE)

Poly­(A) integrity CQA was assessed by CGE using a 5200 Fragment Analyzer System (Agilent, United States).^52^ Fragment separation and detection were performed with the DNF-471 RNA Kit (15 nt) (Agilent), which contains an RNA separation gel, dsDNA inlet buffer, TE rinse buffer, intercalating dye, RNA diluent marker (15 nt), an RNA ladder ranging from 200 to 6000 nt, and a capillary conditioning solution. A FA 12-Capillary Array Short, 33 cm (Agilent), was employed for the analysis.

Poly­(A) samples were initially diluted to ∼100 ng/μL, followed by a 1:12 dilution in the RNA diluent marker to achieve a target final concentration of ∼4 ng/μL. Prior to each run, a prerun voltage of 8 kV for 30 s was applied. Capillaries were conditioned with the provided conditioning solution and rinsed twice with a TE buffer. Capillaries were filled with separation gel under pressure, and samples were introduced by voltage injection (5 kV for 4 s). Electrophoretic separation was carried out at 8 kV for 45 min. Detection was achieved *via* laser-induced fluorescence, facilitated by the intercalating dye present in the separation gel. The resulting electropherograms were used to evaluate Poly­(A) integrity based on fragment size distribution and fluorescence intensity.

### 2.3 Manufacture of Poly­(A)-LNP Formulations and Verification of their Quality Attributes

To further evaluate the impact of Poly­(A) brand on LNP critical quality attributes, ionizable lipid formulations of SM102-LNPs were composed of SM102/CHOL/DSPC/DMG-PEG2000. All initial lipid stock solutions were prepared in ethanol at 5 mg/mL and combined at a 50:38.5:10:1.5 molar ratio for ionizable lipid/cholesterol/helper/PEG-lipid. Branded Poly­(A) was prepared in DNase/RNase-free water at 1.5 mg/mL, verified by NanoDrop, and diluted in 50 mM citrate buffer (pH 6), which was used as the aqueous phase. The lipid organic phase and Poly­(A) aqueous phases were injected simultaneously into the micromixer at a 3:1 aqueous/organic flow rate ratio and a 15 mL/min total flow rate with an NP ratio of 6:1. The final lipid theoretical concentration after microfluidic preparation was 1.25 mg/mL, with a corresponding theoretical Poly­(A) concentration of 0.055 mg/mL. LNP formulations were purified using spin column centrifugation (100 kDa MWCO). Briefly, LNPs were diluted (1:40) in 1× PBS pH 7.4 and centrifuged at 2000 *×g* at 4 °C. Branded LNPs were further characterized using Zetasizer, RiboGreen, Nanoparticle tracking analysis (NTA), and frit-inlet (FI) asymmetric-flow field-flow fractionation hyphenated with multiple inline detectors (FI-AF4-MD) as previously reported.^16^

Nanoparticle tracking analysis (NTA) of particle size, distribution, and estimated particle concentration was conducted using a NanoSight NS300 system (Malvern Panalytical, UK), equipped with a 488 nm laser, a low-volume flow cell, a sCMOS camera, and an automated syringe driver. Samples were diluted 1:10,000 in PBS (pH 7.4) and measured at 25 °C, with the infusion rate set at 50. Data acquisition involved five 60 s videos per sample at a camera level of 15. Analyses were performed using NTA software (version 3.4.003) with a detection threshold of five. Two independent biological replicates, each with five technical replicates, were analyzed per sample.

FI-AF4-MD analysis was performed using a field-flow fractionation system (Postnova Analytics, Germany) coupled with multiangle light scattering (MALS, PN3621), UV detection (260 nm, PN3242), and dynamic light scattering (Zetasizer Nano ZS, Malvern Panalytical). Separation was carried out using a frit-inlet (FI) channel with a 350 μm spacer and a 10 kDa MWCO regenerated cellulose membrane. LNP samples (20 μL injection volume, 0.5 mg/mL) were injected using a 100 μL loop in triplicate. Elution was performed in PBS (pH 7.4) with an injection flow rate of 0.2 mL/min, a crossflow rate of 0.75 mL/min (exponential decay 0.2), and a detector flow rate of 0.3 mL/min. Data were processed using Nova FFF software (v2.2.0.1), applying the spherical MALS model (32–136°) for drug product LNPs.

Transmission electron microscopy with negative staining was used to image LNP formulations for their morphology, in tandem with the AF4 shape factor ratio. Copper grids with carbon film 400 were used (Agar Scientific, AGS160-4) and glow-discharged prior to sample application. A volume of 10 μL of LNP suspension was applied to the grid and incubated for 15 min. Excess sample was removed by filter paper adsorption, and the grid was rinsed once with dH_2_O, followed by a 5 min fixation with 2% glutaraldehyde. Samples were then washed three times with dH_2_O (30 s per wash) and 2% uranyl acetate applied for 5 min. Excess dye was removed by blotting using filter paper, and the grids were left to dry at ambient temperature for 15 min. Samples were imaged using a JEM transmission electron microscope operated at 120 kV, at 30,000, 80,000, and 150,000× magnifications. LNP circularity was quantified from images acquired at the 30,000× magnification in ImageJ software (v.1.8.0).

Each formulation was named A-LNPs, B-LNPs, and C-LNPs as per the Poly­(A) branded manufacturer. Comparative statistical analyses were carried out to correlate the Poly­(A) drug substance with the LNP drug product critical quality attributes.

### 2.4 Statistical Analysis

The corresponding mean ± standard deviation (SD) was calculated for all experiments with a minimum of two independent and two technical replicates unless otherwise stated. A one-way Analysis of Variance (ANOVA) was performed to highlight the statistical significance of comparative analytical data using Dunnett and Tukey tests.

Statistical analyses were performed using Minitab version 20.4 software. Data were graphed using Origin Pro (version 9.9.0.220).

## 3 Results

### 3.1 Electrical Asymmetrical-Flow Field Flow Fractionation (EAF4)

To our knowledge, for the first time, we have developed an EAF4 method to evaluate Poly­(A) attributes multiplexed with inline detectors. The benefit of using EAF4 is the ability to simultaneously profile molecular size and charge properties. Samples formulated in citrate pH 4 buffer were not analyzed due to prior observations of irreversible gelation, which could block FFF membranes. We used an electrical modality in tandem with a flow-based external field to separate Poly­(A) fractions according to their surface charge and diffusion-based size parameters. Since RNAs are inherently anionic, we performed separations under neutral and a positive current (reverse polarity) to examine separation enhancement using an inline hyphenated UV detector at 260 nm. The electrophoretic mobility and resultant zeta potential of separated fractions were calculated from electrical and flow-based separation fields.

EAF4-based separation of Poly­(A) drug substances achieved a >92% recovery (Table 1) from the developed method under neutral and positive current separation conditions, in line with current flow field-flow fractionation ISO standards.^51^ Across all buffer combinations and branded manufacturers of Poly­(A) examined, the application of a positive electrical current in the EAF4 channel enhanced the separation of Poly­(A) relative to neutral current (0 mA) conditions. This demonstrates an enhanced size and surface charge-based separation pipeline through the elution time shift of ∼1.2 min for Brand A, ∼0.5 min for Brand B, and ∼1.7 min for Brand C (Table 1). The UV peak FWHM decreased with the application of the positive current, further signifying enhanced separation with a reduction in the peak width of ∼0.6 min for Brand A, ∼2.2 min for Brand B, and ∼5.2 min for Brand C (Figure 1 and Table 1). With enhanced separation, the UV_260_ signal increased across all brands, with Brand C producing the highest increase at 159.0%, Brand B producing 55.6%, and Brand A producing 8.7%. The purity ratio was calculated as the ratio of the UV peak FWHM at 260 and 280 nm (Figure 1).

**Table 1:** EAF4-MD Poly­(A) Elution Parameters (*n* = 3) ns = No Significant Difference between Buffer Elution Times under *Neutral and ^‡^Positive Conditions Using Tukey’s Test

Brand	Buffer	Current (mA)	Elution time (min)	Peak width (min)	Recovery (%)
Brand A	PBS (pH 7.4)	Neutral (0.0)	24.5 ± 0.2*	9.8	93.6 ± 0.2
		Positive (+1.0)	23.2 ± 0.8^‡^	9.3	94.5 ± 1.9
	Citrate pH 6	Neutral (0.0)	24.4 ± 0.3*	9.9	95.6 ± 2.9
		Positive (+1.0)	23.3 ± 0.5^‡^	9.3	95.3 ± 4.2
Brand B	PBS (pH 7.4)	Neutral (0.0)	25.3 ± 0.2*	6.6	92.3 ± 2.2
		Positive (+1.0)	24.8 ± 0.1^‡^	4.1	95.8 ± 2.2
	Citrate pH 6	Neutral (0.0)	25.3 ± 0.2*	6.2	93.9 ± 1.2
		Positive (+1.0)	24.9 ± 0.1^‡^	4.1	95.2 ± 1.3
Brand C	PBS (pH 7.4)	Neutral (0.0)	27.1 ± 0.2*	8.5	95.0 ± 1.6
		Positive (+1.0)	25.4 ± 0.1^‡^	3.2	98.4 ± 0.8
	Citrate pH 6	Neutral (0.0)	27.1 ± 0.3*	8.4	94.8 ± 2.2
		Positive (+1.0)	25.5 ± 0.1^‡^	3.2	98.6 ± 1.8

**Figure 1:**
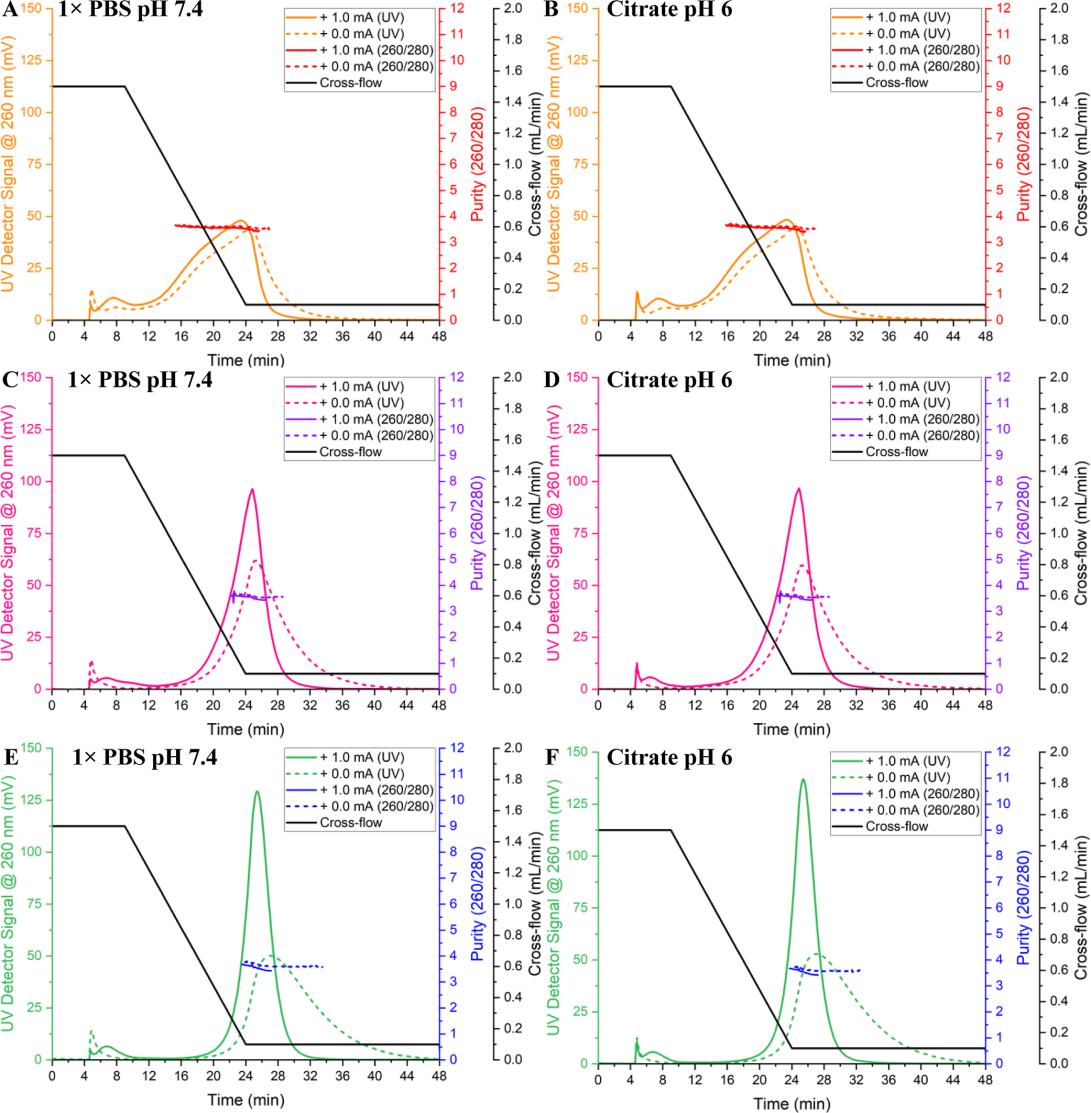
EAF4-UV fractogram traces using 0.0 mA and +1.0 mA currents to separate Brand A Poly­(A) in (A) PBS and (B) citrate pH 6. Brand B (C) PBS and (D) citrate pH 6. Brand C in (E) PBS and (F) citrate pH 6. Poly­(A) was injected at 5 μg. UV elution profiles *n* = 3; purity ratio (260/280) *n* = 2.

Under neutral separation conditions, no significant differences were observed between PBS and citrate pH 6 buffer elution times, highlighting an increased channel eluent buffer dilution factor. The Poly­(A) brands eluted in ascending order of elution time were A < B < C, indicating Brand A Poly­(A) is primarily composed of lower-molecular-weight species and chain lengths in comparison to Brand B and Brand C. Throughout analysis, purity values remained constant with the application of a positive change channel, indicating that Poly­(A) samples from branded manufacturers were of similar purity (>3, Figure 1).

MALS and DLS signals associated with EAF4 samples were low in signal strength; therefore, Poly­(A) enhanced direct injection was utilized, negating a separation field resulting from higher channel dilution under EAF4-based separation conditions. Poly­(A) samples were increased to 12.5 μg from EAF4, separated by a 5 μg injection concentration. Since there was no significant difference between elution times of the Poly­(A) buffer type under neutral conditions, PBS (pH 7.4) buffer was used for Poly­(A) dilution prior to direct injection at the higher concentration.

Poly­(A) elution times remained consistent across samples due to channel direct injection in the absence of external cross-flow and applied electrical fields (Figure 2A,C,E). However, the MALS-90° elution profiles (Figure 2A,C,E) revealed clear differences in detector intensities across equivalent injected concentrations of the branded Poly­(A) drug substance. Brand A produced the lowest detector signal, followed by Brand B, with Brand C exhibiting the highest intensity. These elution profiles indicate underlying chain length size and size distribution differences within each Poly­(A) branded manufacturer, with lower size and higher size distributions producing lower-intensity, broader elution profiles (Brand A), in contrast with higher size and lower size distributions producing higher intensity and narrower elution profiles (Brands B and C, respectively).

**Figure 2:**
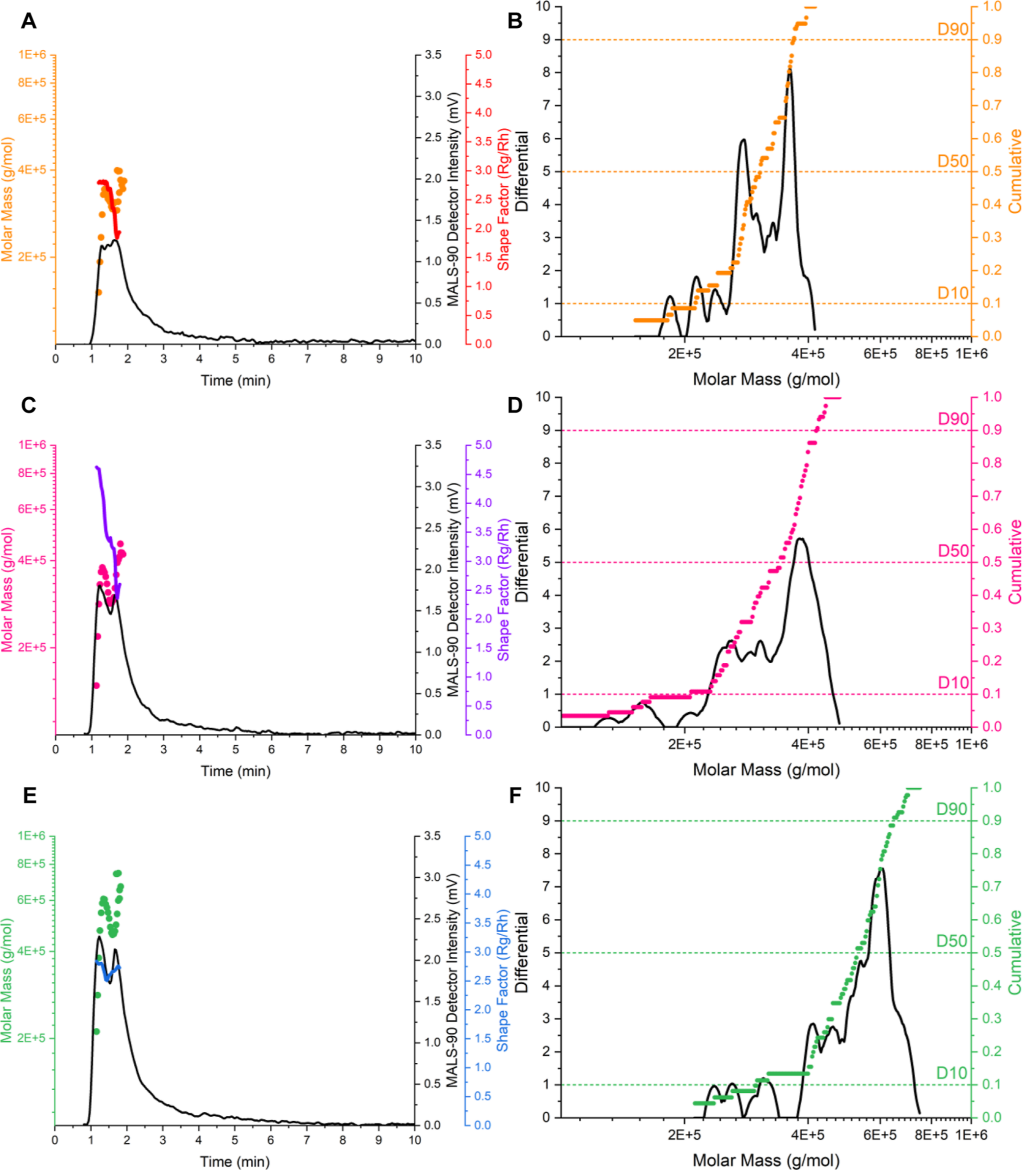
Direct injection (12.5 μg) Poly­(A) evaluation using Brand A Poly­(A) (A) MALS and (B) molar mass distribution, Brand B Poly­(A) (C) MALS and (D) molar mass distribution, and Brand C Poly­(A) (E) MALS and (F) molar mass distribution for 1× PBS pH 7.4. Molar mass distributions integrated at the FWHM MALS-90° detector signal. *n* = 3.

Poly­(A) size and size distribution trends were further reflected in molar mass distribution profiles (Figure 2B,D,F), which highlighted that branded manufacturer molar mass size increased from Brand A through Brand B and Brand C. However, molar mass distributional span and EAF4 PDI (Table 2) showed contrasting nuanced trends, with span values increasing from Brand A to Brand B and then Brand C. EAF4 PDI values showed increasing polydispersity from Brand A to Brand C, followed by Brand B. These contrasting differences in chain length and size dispersity could be due to the direction of injection mode, with the inherent lack of separation in resolving key chain length and size subpopulations.

**Table 2:** EAF4 Enhanced Direct Injection Molecular Weight and Molar Mass Distribution of Poly­(A) Brands Diluted in PBS (pH 7.4), *n* = 3

Brand	Extinction coefficient (mL/g*cm)	Molecular weight (Da)	Molar mass *D*10 (g/mol)	Molar mass *D*50 (g/mol)	Molar mass *D*90 (g/mol)	Span $\left(\frac{D90-D10}{D50}\right)$	EAF4 PDI
Brand A	43.6	297,467	212,558	304,914	367,905	0.51	1.06
Brand B	40.8	315,867	207,512	342,946	418,276	0.61	1.12
Brand C	40.5	508,200	298,918	528,388	648,662	0.66	1.10

Overall, the FFF direct injection data align with EAF4-UV findings, emphasizing manufacturer-specific differences in Poly­(A) CQAs. Molecular weight and molar mass distributions (*D*10, *D*50, *D*90) confirm that Brand A produced consistently lower-molecular-weight species, followed by Brand B, which produced intermediate sizes, and Brand C, which produced the highest size and the lowest polydisperse sample of the three analyzed brands. Estimated Poly­(A) chain lengths were calculated from molecular weight data and molar mass distribution profiles using the expected molecular weight of a single adenylic acid monomer (343 Da) (Table 3). Brand A Poly­(A) produced nucleotide (nt) chain lengths of 620–1073 nts, with Brand B producing nucleotide chain lengths of 605–1219 nts, and Brand C exhibiting nucleotide chain lengths of 871–1891 nts.

**Table 3:** EAF4 Enhanced Direct Injection Estimated Chain Lengths of Poly­(A) Brands Diluted in PBS (pH 7.4), *n* = 3

Brand	Estimated chain length (MW/343)	Estimated chain length *D*10 (*D*10/343)	Estimated chain length *D*50 (*D*50/343)	Estimated chain length *D*90 (*D*90/343)
Brand A	876	620	889	1073
Brand B	921	605	1000	1219
Brand C	1482	871	1540	1891

Further analysis of Poly­(A) morphology was obtained with decreased channel dilution factors due to the direct injection mode, inline DLS *R*_H_ values were obtained, and the shape factor (*R*_G_/*R*_H_) was determined. Shape factor data were averaged over the direct injection peak FWHM to produce averages of 2.5 (Brand A), 3.5 (Brand B), and 2.7 (Brand C) (Figure 2), signifying random coil (>1.2) shape factor morphologies for Brands A, B, and C, with extended spatial structural conformations typically associated with polymers. Morphological ratios can provide deeper insights into structural conformations and manufacturer impact with different molecular weight chain lengths.

Lastly, using the EAF4 pipeline in positive and neutral separation conditions, the charge distribution profile of Poly­(A) was determined, where Poly­(A) electrophoretic mobility and zeta potential were derived to probe the relationship between Poly­(A) structures across different manufacturer sources.

#### 3.1.1 Offline ELS Determination of Electrophoretic Mobility and Zeta Potential

Further insights into Poly­(A) zeta potential distribution were determined using conventional offline electrophoretic light scattering and derived orthogonally from high-resolution EAF4-based separation hyphenated with UV.

All charge-related parameter data obtained from ELS and EAF4 consistently produced negative electrophoretic mobility and zeta potential values due to the inherent anionic profile of RNA.

Zetasizer ELS measurements highlight that with increasing brand chain length, buffer-averaged electrophoretic mobility decreased to more negative values: Brand A (−0.35 μm·cm/(V s)), Brand B (−0.45 μm·cm/(V s)), and Brand C (−1.05 μm·cm/(V s)) (Table 4). The calculated zeta potential also followed equivalent, buffer-averaged results with increased chain length producing more negative surface charges. Variation between individual buffers could reflect counterion diffusion and partial neutralization of anionic phosphate backbone charges and differences in final solution ion concentrations from water dilution, with PBS samples producing approximately 10 mM sodium chloride concentration recommended for zeta potential analysis.^53,54^ EAF4 analysis showed increased consistency in electrophoretic mobilities between buffers, showing high channel dilution and diluting the impact of storage/manufacture buffer on measured measurements. The consistency is further reflected in zeta potential measurements, which were derived from electrophoretic mobility calculations.

**Table 4:** Electrophoretic Mobility and Zeta Potential of Poly­(A) Determined Using ELS (*n* = 2, ± SD) and EAF4-UV (*n* = 3, ± SD)

	ELS			EAF4-UV		
Brand	Buffer	Electrophoretic mobility (μm cm/(V s))	Zeta potential (mV)	Eectrophoretic mobility (μm cm/(V s))	Electrophoretic mobility *R* ^2^	Zeta potential (mV)
Brand A	PBS (pH 7.4)	–0.3 ± 0.2	–3.5 ± 2.4	–0.2 ± 0.1	0.9720	–2.4 ± 1.0
	Citrate pH 6	–0.4 ± 0.4	–5.5 ± 5.2	–0.2 ± 0.1	0.9853	–2.8 ± 1.0
Brand B	PBS (pH 7.4)	–0.2 ± 0.2	–2.5 ± 2.6	–0.1 ± 0.0	0.9965	–1.1 ± 0.5
	Citrate pH 6	–0.7 ± 0.4	–9.4 ± 5.6	–0.1 ± 0.0	0.9876	–1.1 ± 0.5
Brand C	PBS (pH 7.4)	–1.1 ± 0.4	–14.1 ± 5.3	–0.3 ± 0.0	0.9975	–3.7 ± 0.4
	Citrate pH 6	–1.0 ± 0.4	–12.2 ± 5.0	–0.3 ± 0.0	0.9992	–3.3 ± 0.3

Our results in Table 4 demonstrate that switching the buffer from PBS to citrate results in variations in the calculated zeta potential values. This trend was observed with Brands A and B, whereas Brand C deviated by exhibiting a decrease in calculated zeta potential from PBS to citrate buffer. These changes could be due to Poly­(A) conformational differences within citrate buffer, with Brand C having the highest molecular weight species and estimated chain lengths of the three tested Poly­(A) vendors. Statistical analysis using a Tukey post hoc test revealed no significant differences in calculated zeta potentials between PBS and citrate buffers for ELS and EAF4 systems, except for Brand B under ELS conditions, which showed a statistically significant change. Differences between manufacturer brands were less clear using EAF4 as Brands A and C produced similar electrophoretic mobility and zeta potential values, whereas Brand B produced the lowest electrophoretic mobility and zeta potential values. EAF4-MD electrophoretic mobility data showed a significant linear correlation between 0.0 mA neutral and +1.0 mA applied currents, showcased by *R* ^2^ > 0.97 across all electrically separate Poly­(A)­s (Table 4), highlighting a high quality of fit, producing reliable electrophoretic mobility and zeta potential measurements for associated electrically separated Poly­(A) samples. EAF4 data provided deeper insight into charge-based distribution profiles of branded samples, highlighting further manufacturer-based impact on Poly­(A) CQAs.

### 3.2 Analytical Size Exclusion Chromatography

Analytical size exclusion chromatography (aSEC) was used as an orthogonal separation technique to further quantify the impact of Poly­(A) brand on critical quality attributes.

Chromatogram profiles for aSEC-UV data highlight singular monomodal peaks across all Poly­(A) brands analyzed, with Brand C producing the earlier retention time (9.9 min), followed by Brand B (10.3 min) and Brand A (10.6 min) (Figure 3A). Smaller particle sizes and Poly­(A) molecular weights were observed with multimodal peaks for Brands A eluting between 14 and 22 min and Brand B eluting between 19 and 22 min (Figure 3B). UV traces signified that Brand A contains a larger MW and chain length distribution, followed by Brand B and Brand C, characterized by monodispersed MW and chain lengths. aSEC-UV data further verified EAF4-UV elution profiles. A key limitation of aSEC was reduced sample interaction with the stationary phase and AF2000 system pressure limit (15 bar), indicated by early elution within the first column volume (∼25 min). Despite this, measurable size-based separation was still achieved. aSEC-MALS successfully detected Poly­(A) samples at lower concentration than EAF4 direct injection, producing retention time trends consistent with UV profiles, confirming effective, albeit, limited separation.

**Figure 3:**
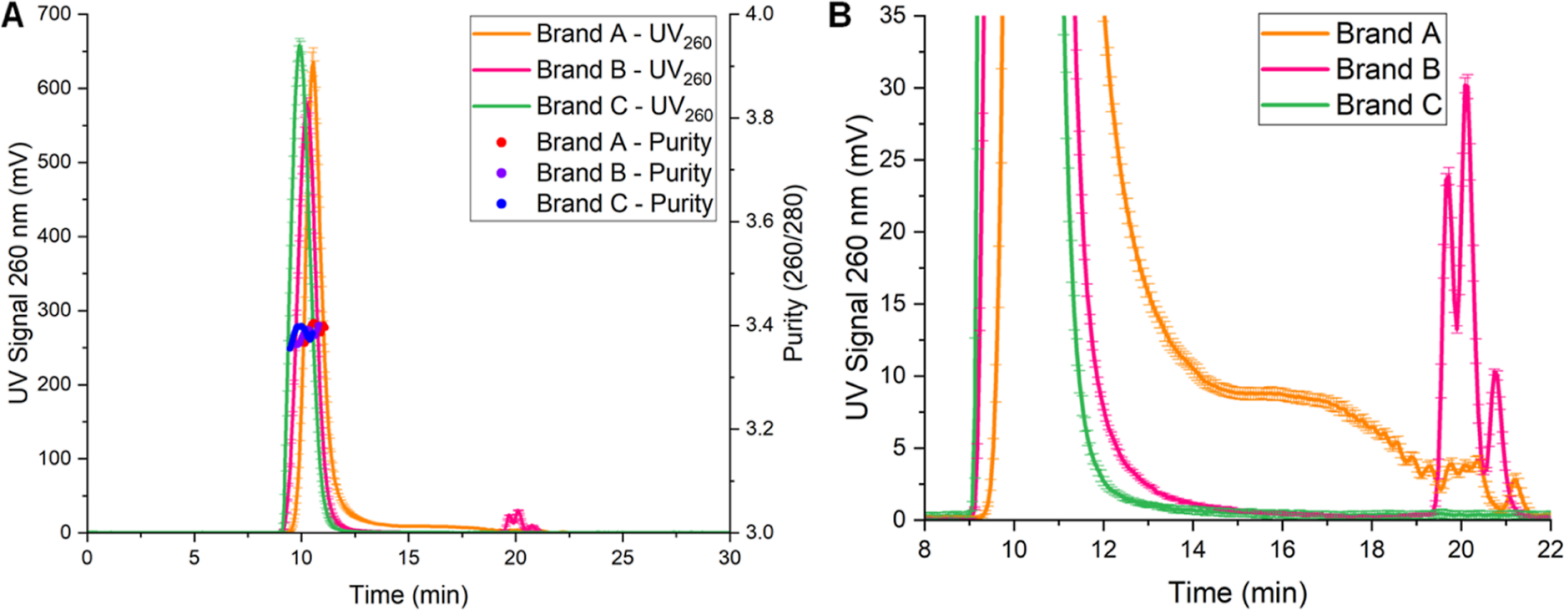
SEC-UV analysis of branded Poly­(A) drug substances: (A) 260 nm absorbance and purity (260/280 FWHM); (B) focused UV detector separation profiles (*n* = 2 ± SD).

To further confirm EAF4 and aSEC results, capillary gel electrophoresis coupled with fluorescence was utilized to separate and quantify Poly­(A) vendor-specific chain length distributions and Poly­(A) integrity. Associated results highlight RNA ladder distribution, Poly­(A) branded samples, and positive control eGFP mRNA separation (Figure S4). CGE findings additionally confirm overall high-level Poly­(A) chain length and molecular weight polydispersity within each branded vendor. CGE results also demonstrate a low signal intensity for the Poly­(A) in comparison to the GFP positive control mRNA (Figure S4). These findings are in agreement with A < B < C in terms of chain length and molecular weight distributions.

Analysis of Poly­(A) MW trends by aSEC-MALS confirmed findings from EAF4-MALS direct injection data that Brand C produced the highest MALS-90° signal and estimated molar mass distribution species across the MALS-90° FWHM profile. An equivalent trend was observed with Brand B and Brand A, in descending order of MALS signal and molar mass distribution (Figure 4). SEC-UV confirmed high to low size-based separation, while unexpected fluctuations were noticed in the molar mass distributions of MALS-90° FWHM profiles are likely due to poor half-maximum peak signal intensity (<1.25 mV), in tandem with lower flow rates, lower injection concentrations, and lower stationary phase interaction than EAF4 separation and direct injection modes. Purity estimations (260/260) were 3–4, highlighting a similar high-quality Poly­(A) sample irrespective of the Poly­(A) brand (Figure 3A), confirming EAF4 values.

**Figure 4:**
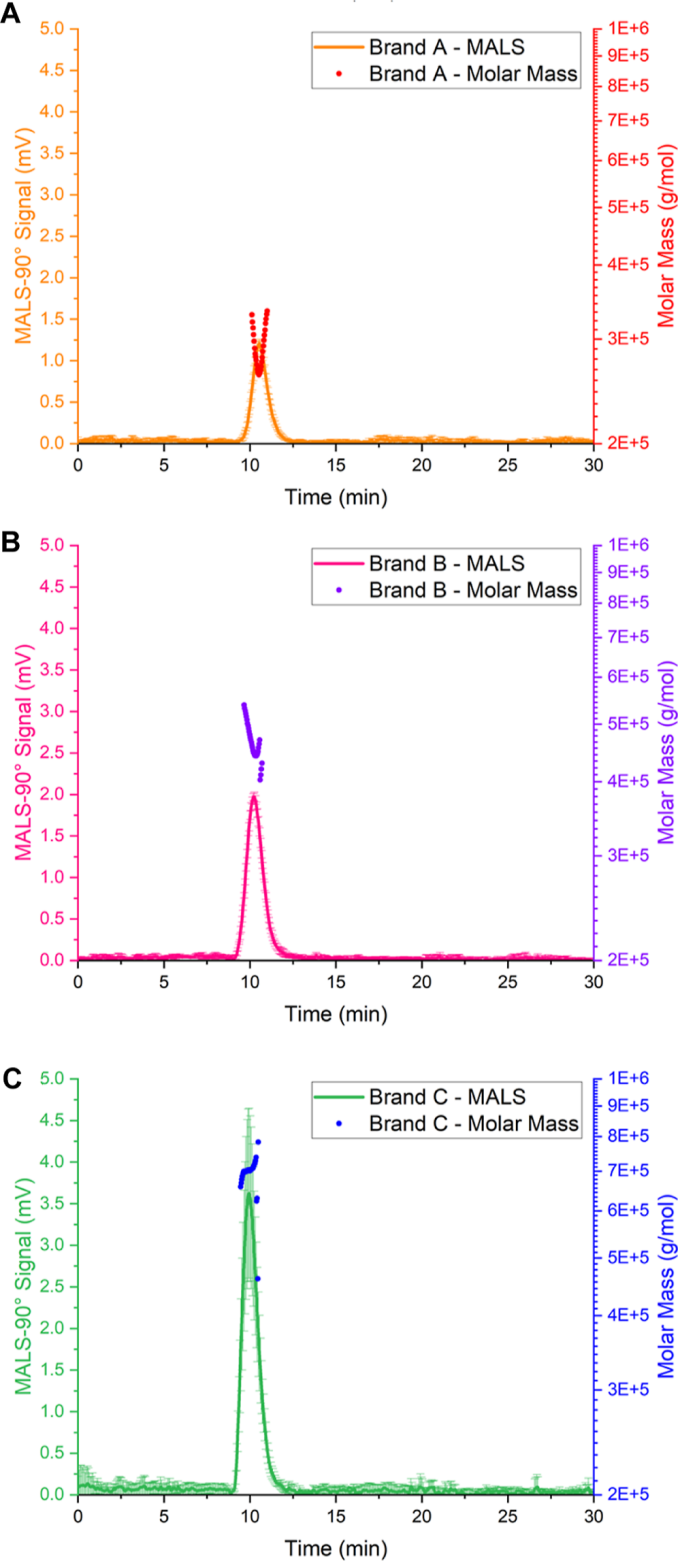
Corresponding aSEC-MALS traces of (A) Brand A, (B) Brand B, and (C) Brand C Poly­(A) (5 μg injection conc) (*n* = 2 ± SD).

### 3.3 Impact of Poly­(A) on Lipid Nanoparticle Formulation Critical Quality Attributes

To further quantify the impact of Poly­(A) molecular weight species and polydispersity on LNP CQAs, we encapsulated each Poly­(A) in an ionizable SM-102 lipid nanoparticle (LNP) formulation.

Initial CQA analyses highlighted similar *z*-average and size distributions between branded Poly­(A) manufactured LNPs, with A-LNPs and B-LNPs producing the smallest (*z*-average) and distribution-based sizes, while C-LNPs produced the largest *z*-average (68.5 nm) and distribution-based sizes (73.1 nm) (Table 5). No significant differences were noted between LNP drug product DLS-measured *z*-average and distributional sizes when comparing the manufacturer-branded Poly­(A) drug substance, despite distinct differences in Poly­(A) manufacturer molecular weights. PDI, zeta potential, and Poly­(A) encapsulation efficiency data produced equivalent results, highlighting the consistency of using standardized formulation and purification processes (Table 5). Poly­(A) mass balance recovery results differed by 18% across the formulations, with A-LNPs producing the lowest recovery at 82.3% and B-LNPs with 97.7%.

**Table 5:** Branded Poly­(A)-LNP Formulation Size Attributes, Measured by DLS, ELS, and RiboGreen Assay for Poly­(A) Encapsulation Efficiency (% EE) and Mass Balance Recovery (% MB) (*n* = 3 ± SD)

	Zetasizer (DLS/ELS)			RiboGreen Assay		
LNP	*Z*-average (*d* nm)	Distribution size (nm)	PDI	*Z*-potential (mV)	% EE	% MB
Brand A	65.7 ± 7.1	71.6 ± 7.5	0.12 ± 0.05	–3.1 ± 1.9	99.4 ± 0.8	82.3 ± 6.8
Brand B	65.9 ± 6.9	71.1 ± 7.5	0.12 ± 0.05	–3.8 ± 2.9	99.1 ± 1.0	97.7 ± 12.0
Brand C	68.5 ± 6.6	73.1 ± 7.2	0.13 ± 0.07	–3.4 ± 2.3	98.9 ± 1.3	97.0 ± 8.3

To further explore the observed differences in Poly­(A) LNP mass balance across brands, the RiboGreen assay was employed using calibration curves generated with each Poly­(A) sample in assay buffer, alongside an rRNA standard, to assess both the concentration accuracy and RNA integrity within the assay. All Poly­(A) samples showed strong linear responses (Figure S5) with high correlation coefficients (*R* ^2^ > 0.98) across both Triton X-100 and TE buffer conditions (Table S3), confirming assay compatibility. No significant differences in calibration linearity were observed between the Poly­(A) drug substances within each buffer group, apart from the rRNA standard. While all Poly­(A) brands produced consistent fluorescence profiles, differences in relative fluorescence intensities across equivalent concentration levels were identified (Tables S4 and S5) with up to a 66% difference between Brands A and C, 33% between Brands B and C, and no significant difference between Brands A and B. These results confirm that Poly­(A) is fully compatible with the RiboGreen assay for total and unencapsulated RNA quantification and suggest that observed mass balance discrepancies across LNP formulations likely stem from formulation-specific or structural factors, rather than incompatibility with the assay.

After investigating mass balance differences, Nanoparticle Tracking Analysis (NTA) was used to deepen our understanding of the size distribution profile of branded Poly­(A) LNPs in higher resolution than DLS.

NTA demonstrated particle-by-particle tracking, highlighting key similarities between formulations such as DLS *z*-average and PDI values. Poly­(A)-LNP formulations produced highly monodispersed particle size distribution profiles, characterized by a monomodal peak (Figure 5). Mean LNP sizes were highly similar at ∼67 nm across all formulations, with corresponding mode sizes ∼61 nm (Table S6). Formulation monodispersity was also confirmed through distributional span data, with each formulation producing similar values of 0.68 (A-LNP), 0.71 (B-LNP), and 0.73 (C-LNP) (Table S6). Using spin column purification, equivalent estimated particle concentrations were achieved across all three LNP formulations (∼2.6 × 10^11^ particles/mL) (Table S6).

**Figure 5:**
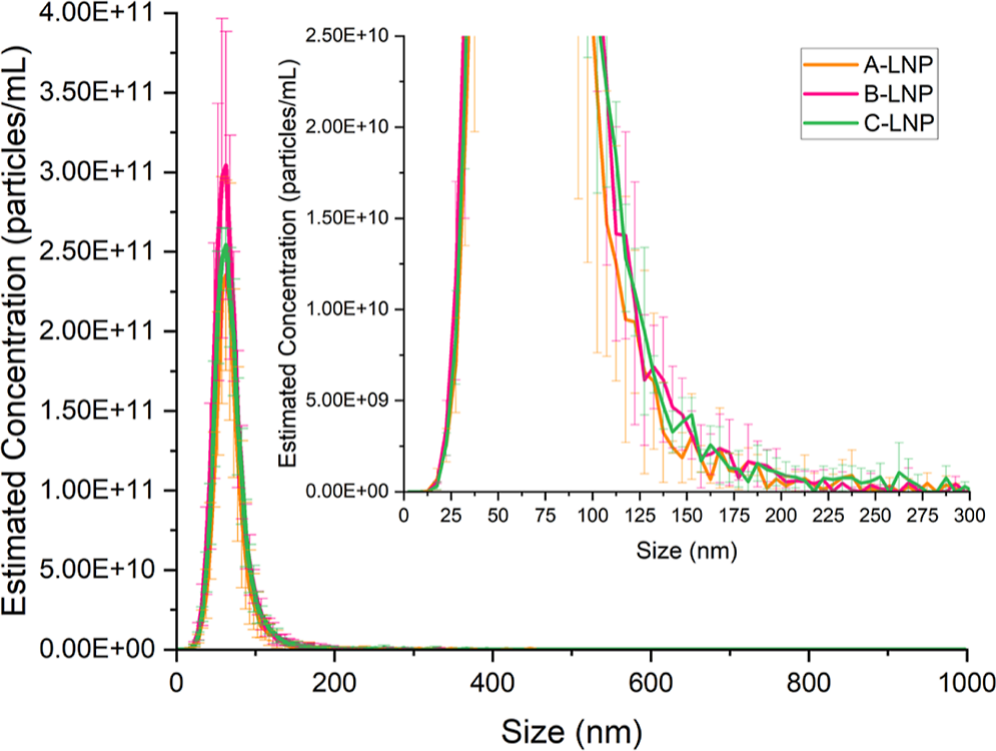
Nanoparticle Tracking Analysis particle size distribution profiles of branded Poly­(A)-LNPs using raw data exports (*n* = 3, ± SD).

Zetasizer and NTA data showed highly similar CQAs across the Poly­(A) A-LNP, B-LNP, and C-LNP formulations. Frit inlet asymmetric-flow field-flow fractionation was used to profile formulation characteristics, enabling size and morphological evaluation.

Our previously developed LNP-based AF4 separation method^16^ was used for LNP formulations, resulting in high percentage recoveries (>88%) and high MALS 90° detector signals (>40 mV) (Figure 6 and Table S7). A and C-LNPs produced channel elution times of 22.3 min, corresponding to a mode radius of gyration (*R*_G_) of 24.7 and 24.6 nm, respectively. B-LNPs eluted earlier at 21.9 min and produced mode *R*_G_ sizes of 24.2 nm (Table S7). A-LNPs produced a MALS 90° signal of 43.3 mV, B-LNPs 60.0 mV, and C-LNPs at a signal intensity of 59.2 mV (Figure 6). Separated LNPs (Figure S6) have been shown to contain Poly­(A) drug through overlapping UV (260 nm) trace quantifying drug absorbance and MALS-90° trace analyzing LNP scattering, highlighting drug loading. Cumulative distributional data (Figure 6D–F) highlight further formulation monodispersity through narrow ranges of radius of gyration (6 nm), hydrodynamic radius (7 nm), and shape factor (0.06). B-LNPs produced the smallest radius of gyration distributions, denoted by a left shift to a smaller size range (Figure 6D), whereas A-LNP and C-LNP produced similar *R*_G_ size distributions, indicated by overlapping profiles (Figure 6D). Hydrodynamic radius distribution (Figure 6E) showed similar sizes from *R*_H_10–50 distributional values across three branded Poly­(A) formulation types, from *R*_H_50–90 to *R*_H_10–50. Brand C-LNPs produced larger sizes of LNPs, with a right shift toward a larger size range. Brand B and C-LNPs produced similar trending shape factor values across the FWHM distribution, whereas A-LNPs produced larger shape factor values across the 0.1–0.9 distribution (Figure 6E). Nuanced differences between branded Poly­(A)-LNP formulations’ cumulative distributional *R*_G_, *R*_H_, and shape factor data were compared (Figure 6F–I and Table S7) to evaluate distributional values between formulations. To further confirm AF4 shape factor morphologies, electron microscopy was used to image A, B, and C-LNPs for further insights and comparisons.

**Figure 6:**
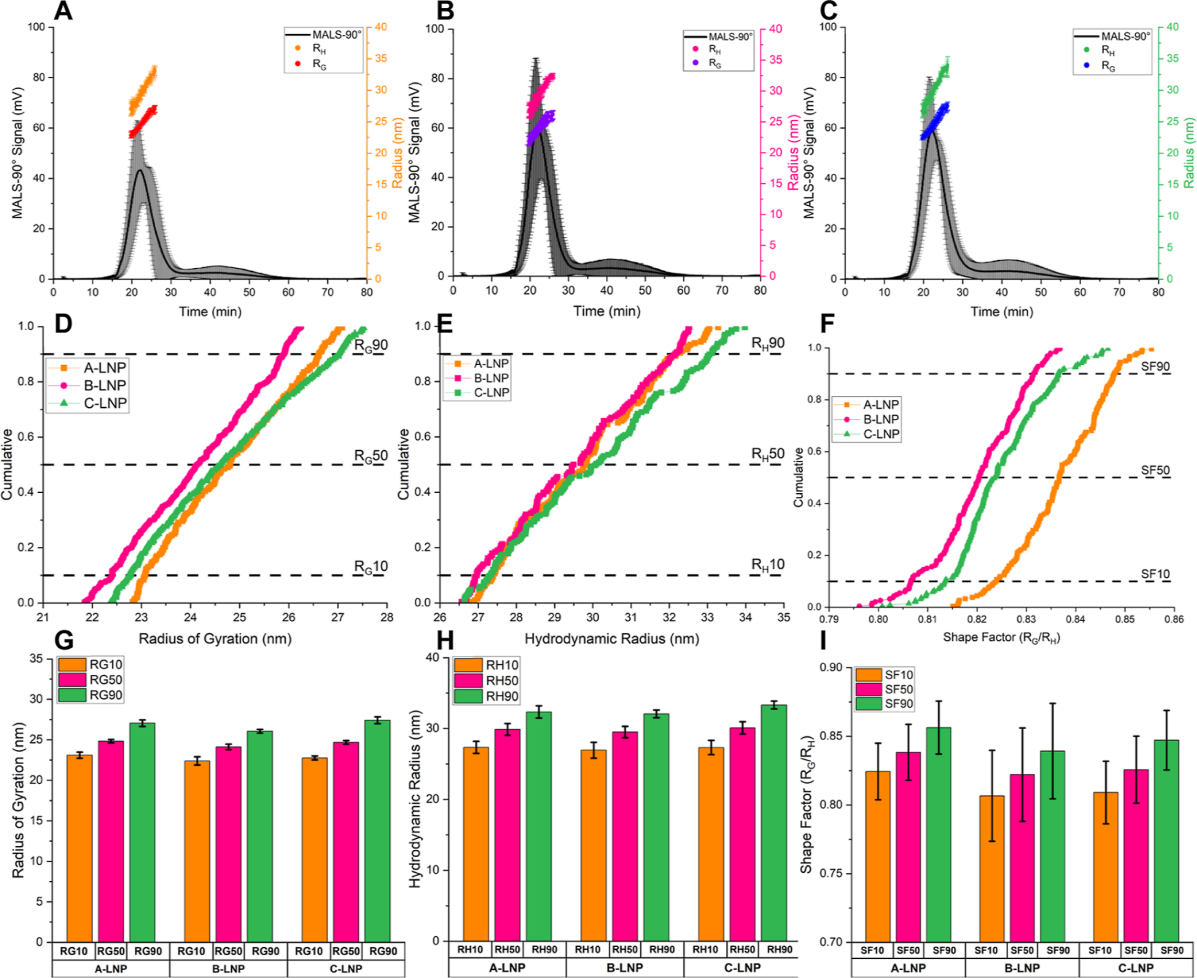
FI-AF4-MD of Poly­(A) LNP formulations. MALS-90° elution profiles with FWHM-integrated radius of gyration and hydrodynamic radii for (A) A-LNPs, (B) B-LNPs, and (C) C-LNPs (*n* = 3 ± SD). Cumulative distributions for (D) radius of gyration, (E) hydrodynamic radius, and (F) shape factor (*n* = 3), with respective 10, 50, and 90 distributional values for (G) radius of gyration, (H) hydrodynamic radius, and (I) shape factor for A-LNPs, B-LNPs and C-LNPs (*n* = 3 ± SD).

Negative stain-TEM proved successful for imaging Poly­(A)-LNPs, where each formulation highlighted deviation from the spherical morphology. Circularity measurements for associated LNP formulations were 0.785 (A-LNPs), 0.785 (B-LNPs), and 0.779 (C-LNPs), respectively. With 1.0 equating spherical morphology, a 22% decrease was determined, departing from a complete spherical assumption. The shape factor derived from each LNP formulation directly aligns with the shape factor data determined for AF4, where 0.775 assumes a perfect sphere with an average SF90 (Table S7) of 0.847, producing a 9.3% increase in shape factor from spherical toward elongated morphologies.

Collectively across LNP analytical pipelines, no significant differences in CQAs between A-C LNP formulations were found, highlighting that differences in the labeled Poly­(A) chain length were not sufficiently large to produce significant differences in LNP CQAs. Correlation between the collective Poly­(A) drug substance and Poly­(A)-LNP formulation CQAs properties were correlated to establish the link between the drug substance parameters and the drug product attributes to further deepen our understanding of pharmaceutical development.

On comparison of drug substance versus drug product matrices and correlation plots (Figure 8), the drug substance matrix shows tight groupings of branded Poly­(A) CQAs, further highlighting differences in drug substances according to the Poly­(A) brand (Figure 8A). The correlation strength drug substance plot further exemplified the relationship between the analyzed CQAs. Strong positive correlations (>0.6) include salt content and EAF4 elution time, SEC retention time, SEC peak number with ELS electrophoretic mobility and ELS zeta potential, EAF4 elution time, and Poly­(A) molecular weight (Figure 8B). Negative correlations (>-0.6) were observed between several parameters, including salt content, ELS electrophoretic mobility, ELS zeta potential, EAF4 elution time with DLS *z*-average, DLS PDI, and between ELS electrophoretic mobility and ELS zeta potential, as well as DLS *z*-average and EAF4 molecular weight (Figure 8B).

**Figure 7:**
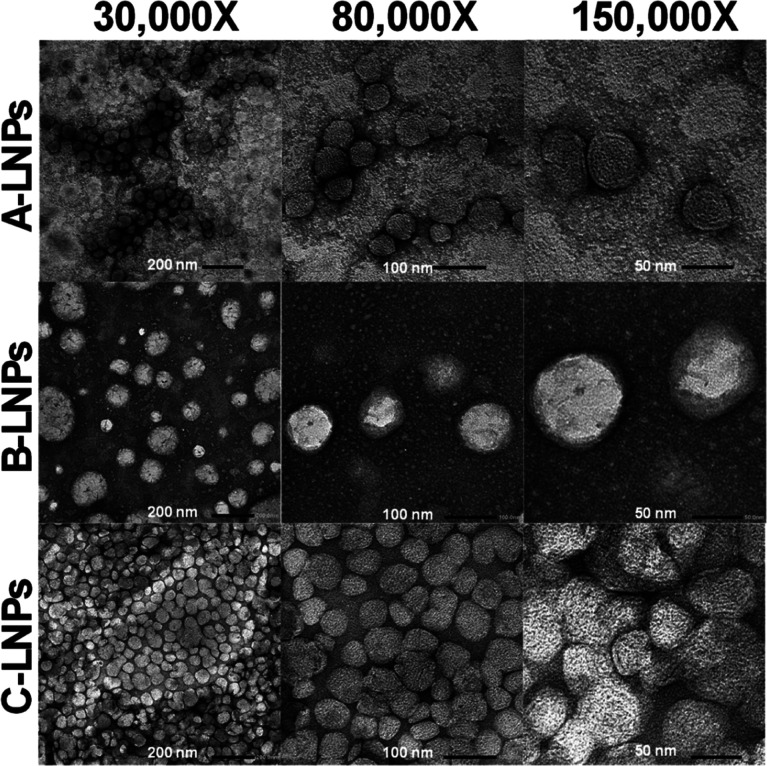
Negative stain transmission electron microscopy of Poly­(A)-LNP batches with increasing magnification. 10 μL of 1.25 mg/mL (formulation concentration) LNPs were imaged, and a minimum of 30 particles were analyzed to obtain circularity data (*n* = 1).

**Figure 8:**
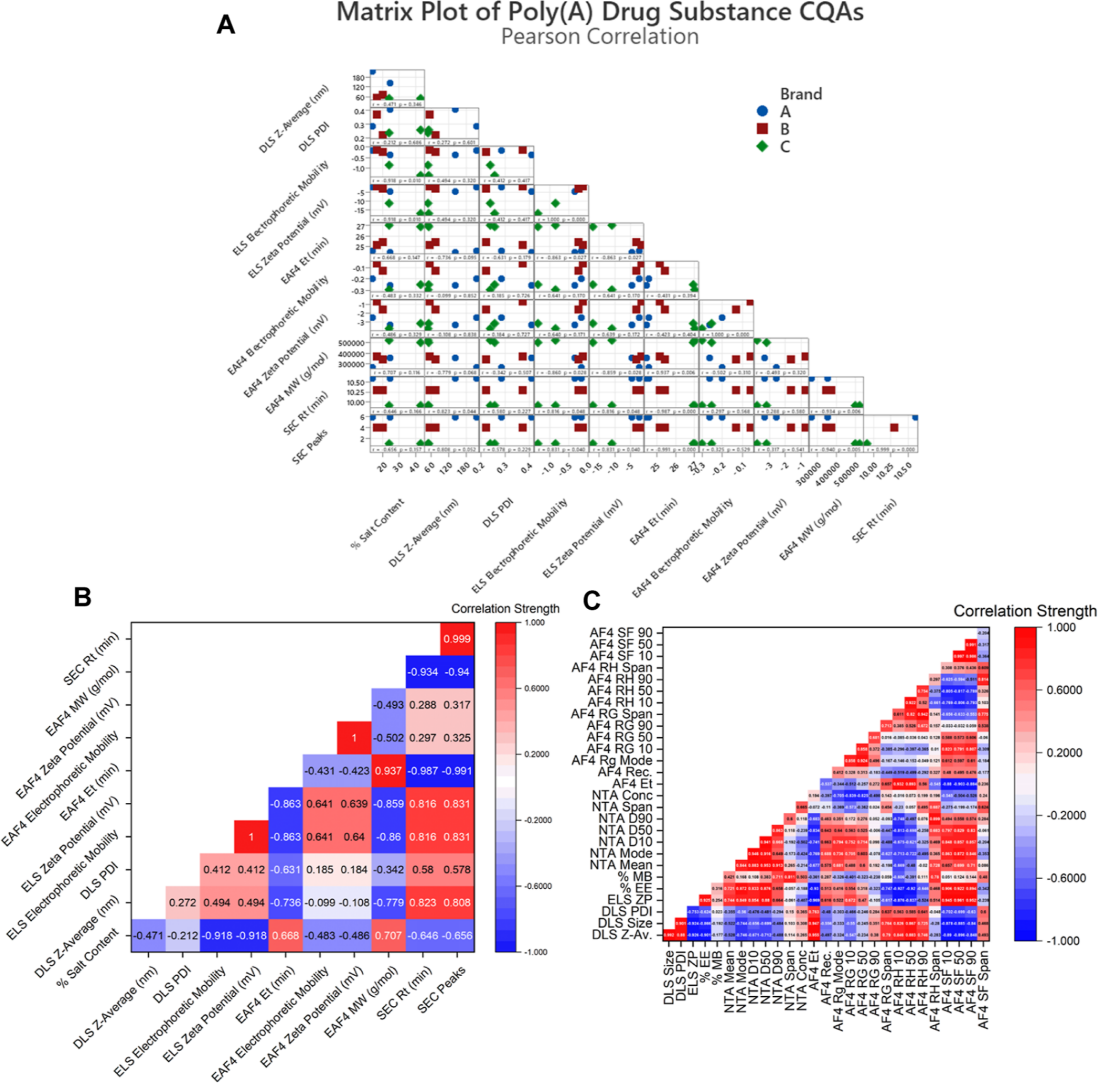
Correlation of Poly­(A) CQAs from the drug substance and drug product. (A) Matrix plot of drug substance CQAs with associated (B) correlation strength heatmap and (C) LNP drug product correlation strength heatmap.

With no significant differences between LNP formulations determined, all LNP technique CQA outputs were combined in a correlation matrix to evaluate the relationships between CQA outputs and their strength (Figure 8C). CQA outputs were split into two plots, with Zetasizer, RiboGreen, and NTA CQA outputs in one plot and all FI-AF4-MD outputs in another plot. Trending tight grouping of the drug substance did not translate to LNPs across all brands when analyzing the matrix plot due to high similarities between the branded Poly­(A)-LNP formulations (Figure S7A).

No formulations produced high batch-to-batch consistency when comparing analytical CQA outputs (Figure S7A). As a result, an increase in strong positive and strong negative correlations was seen between DLS, ELS, RiboGreen, and NTA CQAs. Strong positive correlations (>0.6) were noted between DLS CQAs (*z*-average, size, and PDI), NTA CQAs (mean, mode, *D*10, *D*50, *D*90), mass balance, and NTA *D*90 and NTA span measurements (Figure S7B). Strong negative correlations (>-0.6) were observed between ELS zeta potential and DLS CQAs (z-average, size, and PDI), Poly­(A) % EE, and DLS CQAs (*z*-average, size, and PDI (Figure S7B). Neither strong positive nor negative correlations were noted between Poly­(A) % MB and DLS, ELS % EE, or NTA CQAs (mean, mode, *D*10, *D*50, *D*90, conc).

Deeper insights into size and morphology distributions were highlighted through a matrix plot of AF4 CQAs (Figure S7C). Interestingly, even with no statistically significant differences between formulations, CQAs derived from AF4 measurements highlighted differences in correlation strengths between measured distributional outputs (Figure S7D). Strong positive correlations (>0.6) were noted between 24.8% of CQA outputs (Figure S7D). Strong negative correlations (>−0.6) were observed between 12.4% of CQAs, including AF4 elution time and shape factor distributions (SF10, SF50, SF90). 62.8% of AF4 CQA outputs produced neither strong positive nor strong negative correlative relationships.

## 4 Discussion

Within the therapeutic nanomedicine landscape, interests in LNP-based delivery systems are only increasing, while the critical impact of active drug substance remains under-evaluated, causing a gap in the drug substance to drug product evaluative impact. In this study, we compare three commercially available Poly­(A)­s using combinatorial analytical pipelines and evaluate their impact within an LNP-based drug product system using established analytical pipelines to bridge the gap and translate differences in nucleic acid drug substance attributes to LNP drug product.

We used different biophysical techniques to characterize and evaluate potential differences between branded Poly­(A) drug substances to assess their intrinsic critical quality attributes from associated salt content, quantification assay suitability, aggregation probability, size, and size distribution through standardized techniques, novel surface charge separation, and orthogonal chromatographic methodologies, to enable a larger overview of branded drug substances as a collective. Despite clear physicochemical differences between branded Poly­(A) drug substances, LNP formulation mitigates these variabilities, producing consistent and comparable LNPs.

Previous studies^55–58^ have shown that RNAs can form secondary and tertiary higher-order structures using complementary base–pair interactions. However, as Poly­(A) exists as single-stranded RNA, other intermolecular forces, aside from classic base pairing interactions, play a crucial role in driving Poly­(A) potential conformational and physiochemical changes under preformulation conditions relevant to drug delivery systems. Poly­(A) structural conformation has previously been studied in depth using various biophysical analytical techniques, using short-chain Poly­(A),^59,60^ self-association,^61^ and helical conformations,^62^ with complementary polyuridylic acid (poly­(U))^63^ under various pH, temperature, and ionic strength conditions.

The combinatorial approach in our study highlighted salt content and concentration differences (Figure S1 and Table S1). DLS data highlighted poor scattering of Poly­(A)­s through high standard deviations between replicate samples across brands and manufacturing buffers, indicating that DLS cannot be adopted as an analytical technique for drug substance analysis (Figure S2 and Table S2). Gelation noticed during DLS analysis was not highlighted through differences in the diffusion coefficient with dilution in different buffers used during the manufacture of LNPs (Figure S3).

To our knowledge, this is the first report of applying EAF4 to the analysis of nucleic-acid-based drug substances. Optimization of the EAF4 separation resulted in recoveries in excess of 92%, highlighting excellent separation and detection ability using neutral and positive currents in addition to crossflow external separation force fields (Table 1). Poly­(A) samples exhibited enhanced separation with the application of a positive current within the EAF4 channel, highlighted through a shift in retention times, with smaller Poly­(A) chain lengths eluting earlier, followed by larger chain lengths (Figure 1). EAF4 data highlight that the Brand A Poly­(A) drug substance contained a higher dispersity of chain lengths in comparison to the monodispersed, intense, narrow peaks observed with Brand B and Brand C Poly­(A)­s (Figure 1). Comparing Poly­(A) samples diluted in citrate pH 6 and PBS showed no significant differences in elution profiles (Table 1). All Poly­(A) brands tested produced high purity ratios, compared to 260/280, with all brands producing values ∼3.5 over an integrated FWHM elution peak, irrespective of the current applied within the channel (Figure 1). Since EAF4 sample injection into the channel produced high dilution, MALS signals were not recovered from separated Poly­(A) samples; however, enhanced Poly­(A) concentration (12.5 μg) direction injections were used to obtain MALS data for each branded manufacturer (Figure 2 and Table 2) to evaluate and estimate molecular weight and sample molar mass distributions.

Aligning with separation data, direct injection data show that the Poly­(A) molecular weight increased between different Poly­(A) brands, with Brand A having a lower molecular weight and chain length Poly­(A)­s than Brand B and Brand C (Table 3). Brand A produced the smallest MALS signal and thus produced lower overall span/PDI values, whereas Brand B and Brand C Poly­(A) produced higher MALS signals and trended with higher span/PDI values (Table 2), following a converse trend from monodispersed EAF4 separation data. Shape factor (*R*_G_/*R*_H_) data was obtained, producing differing estimated morphologies for each brand with expected random coil (∼1.5); differences could be caused by low inline DLS signals (<50 kcps) (Figure 2), so further investigation would be required for morphological differences between branded Poly­(A)­s as a drug substance.

With the novel use of EAF4, Poly­(A) electrophoretic mobility and zeta potential can be calculated from the use of neutral and positive currents applied to the separation channel and compared to conventional batch-mode offline ELS Zetasizer electrophoretic mobility and zeta potential evaluations (Table 4). Our results reaffirm successful separation with calculated inherent anionic zeta potential of the Poly­(A) branded samples from superior quality electrophoretic mobility results (*R* ^2^ > 0.97). Differences between the Zetasizer ELS electrophoretic mobility and zeta potential (Table 4) could be due to dilution factors within the channel and the contribution of Poly­(A) sample buffer to Zetasizer ELS measurements from potentially higher-concentration samples.

To further confirm EAF4 data, aSEC-UV-MALS was used as an orthogonal separation technique to evaluate Poly­(A) molecular weight species and estimated chain length distributions (Figures 3 and 4). Chromatogram traces from aSEC-UV produced high-purity ratios, aligning with EAF4-UV fractogram traces, confirming EAF4-UV molecular weight and chain length differences between the different Poly­(A) drug substance brands, with Brand C eluting earlier, followed by Brand B and then Brand A, highlighting the expected opposing order from the EAF4-UV results (Figure 3). With aSEC-UV data producing enhanced detector signals, differences in size distributions were noted with Brand A containing higher levels of lower-molecular-weight species, followed by Brand B and C containing a single peak distribution (Figure 3). Additionally, CGE was utilized to further verify the use of AF4 as a novel analytical methodology for profiling molecular weight distribution. Our CGE results are in agreement with previous EAF4 and aSEC results, with high polydispersity observed across all vendors, with increasing chain lengths (A < B < C) (Figure S4). These results demonstrate vendor-specific Poly­(A) distributions across each tested brand.

With differences noted in branded Poly­(A) drug substance molecular weights and associated chain length distributions, the fundamental impact within a lipid nanoparticle delivery system as a collective drug product was evaluated. Standardized ionizable formulations of SM102/CHOL/DSPC/DMG-PEG2K were used to encapsulate branded Poly­(A)­s during microfluidic formulation, and downstream purification and buffer exchange were achieved using centrifugal spin columns and PBS (pH 7.4) buffer. Established LNP analytical pipelines were utilized for evaluation of A-LNP, B-LNP, and C-LNP formulations, including Zetasizer DLS/ELS, RiboGreen, NTA, and FI-AF4-MD techniques.

Initial DLS size and PDI results indicate highly monodisperse LNP formulations (PDI < 0.2) of similar *z*-average and size distributions (60–70 nm). Further drug product similarities were noted with zeta potential (−3.5 mV), % EE (>98%), and % MB (>80%) with no statistically significant differences between A-LNPs, B-LNPs, and C-LNPs (Table 5). Using 100 kDa MWCO spin columns during formulation could selectively remove smaller LNP populations, resulting in comparable size distributions across drug products. This impact would be most evident in A-LNPs, formulated with Brand A Poly­(A), which exhibited a smaller chain length but a broader molecular weight range. The presence of smaller LNPs formed from lower-molecular-weight species would be filtered out during centrifugation, resulting in a higher *z*-average, reduced PDI, and lower overall Poly­(A) recovery in mass balance calculations compared to the more monodisperse B-LNPs and C-LNPs derived from Brands B and C. Further RiboGreen assay analysis highlighted that, despite being from branded manufacturers, all Poly­(A)­s behaved similarly within the assay, proving compatibility and retained integrity (Figure S5 and Table S3). Concentration levels within assay calibration curves showed significant differences between brands; however, independent replicates produced a small standard deviation between measurements, highlighting a narrow data range and a cause for significant differences.

NTA as a single particle tracking approach was used to profile the impact of Poly­(A) input materials on LNP formulations, relating particle Brownian motion to size by using the Stokes–Einstein equation. NTA size distribution profiles exhibited no statistically significant differences between branded Poly­(A) drug product LNP formulations (Figure 5 and Table S6), further emphasizing the enhanced reproducibility of formulation and purification processes within the model LNP formulation utilized. Although NTA remains a high-resolution technique compared to conventional DLS as an ensemble method, NTA does not incorporate separation of samples, which was addressed in this study using the FI-AF4 methodology coupled with inline UV-MALS-DLS.

Outputs from AF4, including *R*_G_, *R*_H_, and shape factor morphology data, were used to further profile A-LNP, B-LNP, and C-LNP formulations (Figure 6, Table S7, and Figure S6). Previous similarity trends between formulations were also realized using AF4 separation and detection methodology, with no statistically significant difference between branded Poly­(A) formulations. Although no statistically significant differences were noted, nuanced differences were determined when comparing formulation cumulative distribution *R*_G_, *R*_H_, and shape factor values (Figure 6 and Table S7). Lower MALS distributional and *R*_G_ span values were observed for B-LNPs, whereas C-LNPs exhibited higher *R*_G_90 sizes, along with a higher *R*_G_ span (Table S7). DLS *R*_H_ sizes produced an equivalent trend, aligning with MALS data that B-LNPs produced lower *R*_H_ (Table S7). Shape factor distributions also followed similar trends, with the lowest values produced by B-LNPs. However, increased shape factor distribution values were produced from A-LNPs, while shape factor span was highest for C-LNPs (Table S7). AF4 shape factor morphology was confirmed by analysis of LNP negative stain-TEM micrographs, where circularity was analyzed to cross-compare orthogonal techniques (Figure 7). AF4 highlighted a 9.3% deviation, and TEM demonstrated a 21.7% deviation from spherical toward elongated LNP morphologies, showing spherical to oval particle shapes.

Critical quality attributes remain key descriptors of the biophysical characteristics of advanced therapeutics and novel nanomedicine quality. CQAs can be further utilized within matrix grouping and correlation analysis to further enhance our knowledge of analytics. This enables a pivot toward deep analytical networking and application of artificial intelligence and machine learning algorithms to process these correlative outcomes and provide improved therapeutics to boost therapeutic translatability. Here, we correlate drug substance CQA outputs (Figure 8A,B) and drug product CQA outputs (Figures 8C and S7A–D) to gain deeper insight into how manufacturer-specific attributes influence both Poly­(A) input material and resultant LNP formulations. The Poly­(A) drug substance matrix showed tight groupings due to differences in molecular weight and chain length distributions when encapsulated into an LNP drug product. These differences were reduced, and drug product formulations produced no significant differences between LNP formulations, highlighting robust formulation and purification methods. While Pearson’s correlations were used to assess relationships between CQA outputs, the number of experimental replicates requires interpretations to be made with caution. These correlations offer preliminary insights, and further confirmation of relationships would require additional experimental replicate testing to enhance the statistical power and robustness. When analyzing the LNP drug product correlation plot (Figure S7), zeta potential plays a key role in colloidal stability through the DLVO theory.^55–57^ Using the correlation plot, LNP zeta potential displayed strong negative correlations with DLS *z*-average, distribution size, PDI, AF4 elution time, and *R*_G_ span, highlighting that as these CQAs increase in value, zeta potential decreases (Figure S7), which overall emphasizes the fundamental impact of zeta potential on overall formulation CQAs and technique analytical outputs. Other fundamental relationships can be viewed with size and size distributions of drug products by comparing DLS *z*-average, DLS size, DLS PDI, NTA mean, NTA mode, NTA distributions (10, 50, 90, span), AF4 *R*_G_ mean, and *R*_G_, *R*_H_, and shape factor distributions (10, 50, 90, span). These results and correlations should be interpreted within the context of the specific Poly­(A) brands and SM-102 LNP formulation conditions used in this study, with further work being a requirement to test lipid/RNA ratios, main ionizable lipid changes, lipid molar ratio changes, storage buffer changes, drug substance sourcing, and sequence specificity.

## 5 Conclusion

Using a combinatorial analytical pipeline, we demonstrated clear differences in the Poly­(A) brands. Brand A exhibited lower molecular weights with broader molar mass distributions, while Brand B showed intermediate properties, and Brand C presented the highest molecular weights with a narrower, monodisperse distribution. Despite these distinctions at the drug substance level, no statistically significant differences were observed across LNP drug products in terms of key CQAs, indicating a limited impact of Poly­(A) molecular weight variability on overall LNP formulation performance. To further explore potential internal LNP structural features, advanced techniques such as electron microscopy, small-angle X-ray scattering, and calorimetry could offer deeper resolution into Poly­(A) packing within LNPs. Importantly, correlated CQA outputs from Poly­(A) drug substance analyses revealed consistent manufacturer-specific grouping across orthogonal methods, reinforcing the need for more analytics within drug substance development. In contrast, LNP drug product CQAs yielded a broader correlation network, highlighting the value of integrating both routine and high-resolution analytics to deepen our understanding of how drug substance characteristics influence the final drug product profile.

## Supplementary Material



## Data Availability

The data which supports the findings of this study can be found at https://doi.org/10.15129/925cb9fa-6b66-4d8c-97cd-752b87941096.

## Notes

Poly­(A) drug substance concentration verification (Figure S1) associated average differences, RSD, linearity, and LOD/LOQ values (Table S1); Poly­(A) DLS size distribution (Figure S2) and *z*-average, PDI, and individual peak sizes (Table S2); Poly­(A) DLS calculated self-interaction parameter (*K*_D_) across different buffers (Figure S3); Poly­(A) orthogonal separation technique CGE profiles (Figure S4), with RiboGreen evaluation of Poly­(A)­s compared with the assay rRNA standard (Figure S5); comparison of Poly­(A) and rRNA standard linearity and LOD/LOQ values (Table S3); statistical comparisons between each Poly­(A) brand using the Triton X-100 curve (Table S4) and TE curve (Table S5); further CQA values derived from NTA (Table S6) and AF4 analyses (Table S7), with AF4 overlapping MALS/UV trace to show particle loading across the AF4 elution profile (Figure S6); and correlation matrices and heatmaps for Poly­(A)-LNP output CQAs (Figure S7) (PDF)
