# Supplementary material for: The Use of Enhanced Analytical Pipelines for the Characterization of Poly(A) and Poly(A)-LNP Formulation Critical Quality Attributes
Source: Mol Pharm. 2025 Oct 30;22(12):7383–99. doi: 10.1021/acs.molpharmaceut.5c00614 (PMC12673581; doi:10.1021/acs.molpharmaceut.5c00614)
Supplement: Supplementary file 1 [file mp5c00614_si_001.pdf]

The Use of Enhanced Analytical Pipelines for the Characterisation of Poly(A) and Poly(A)-LNP Formulation Critical Quality Attributes

Callum G. Davidson[1], Eleni Kapsali[1], Savvas Ioannou[2], Bojan Kopilovic[3], Muattaz Hussain[1], Yvonne Perrie[1], Zahra Rattray[1]*

1. Strathclyde Institute of Pharmacy and Biomedical Sciences, University of Strathclyde, Glasgow, G4 0RE.
2. School of Molecular Biosciences, University of Glasgow, Glasgow, G11 6EW
3. School of Chemical, Materials and Biological Engineering, University of Sheffield, Sheffield, S1 3JD.

*Corresponding author: Zahra Rattray (zahra.rattray@strath.ac.uk)

Keywords: EAF4, Drug Substance, Drug Product, Poly(A), LNP

Supplemental Information

1.1 Verification of Concentration

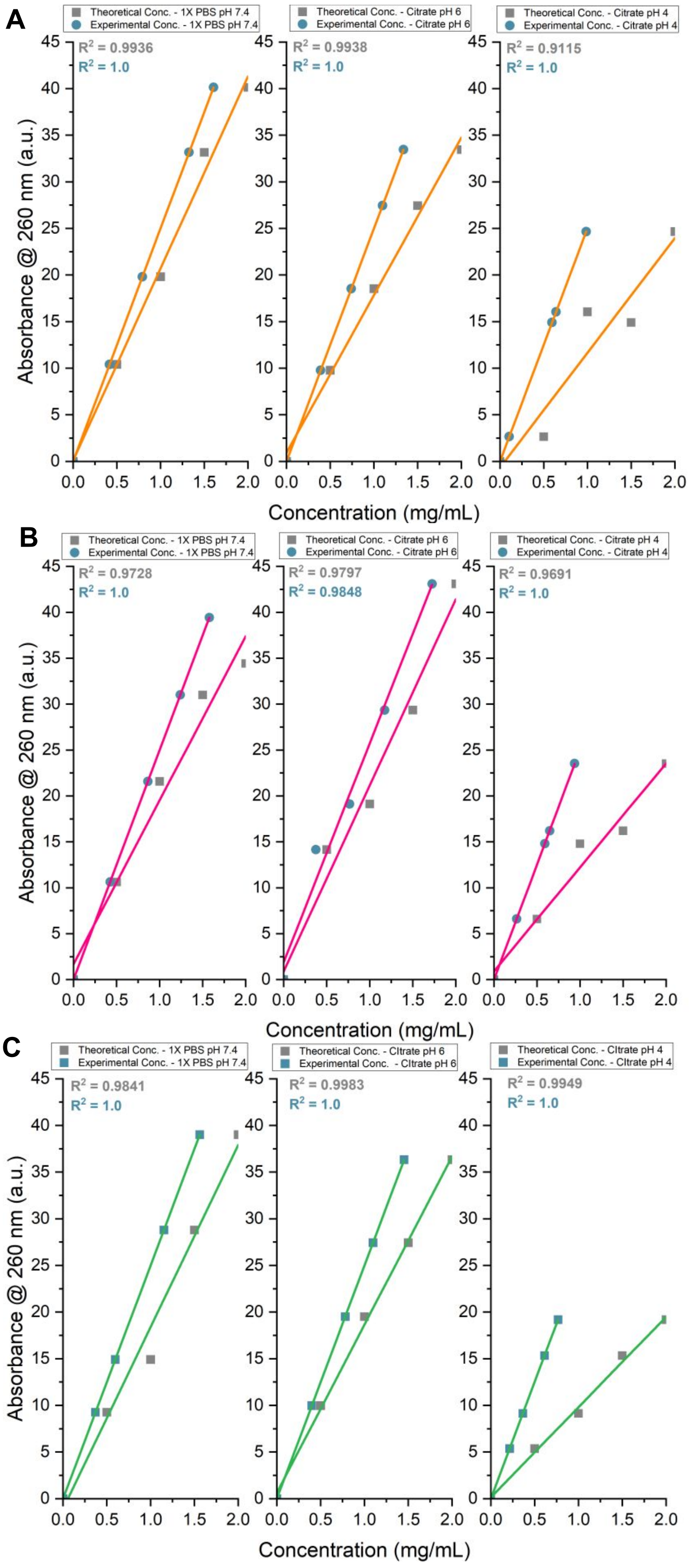

Figure S 1 - Absorbance calibration curves of theoretical concentration versus experimentally calculated concentration of Poly(A) in formulation buffers from A) Brand A, B) Brand B and C) Brand C, as a function of absorbance @ 260 nm. Data represents n=2.

Table S 1 - Average percentage difference between theoretical and experimental Poly(A) concentrations (0.5-2.0 mg/mL)(n=2), linearity (R^2) Limit of Detection (LOD) and Limit of Quantification (LOQ) for Poly(A) in manufacture buffer conditions from NanoDrop linear regression of calibration curves (0.5 mg/mL – 2.0 mg/mL) (n=1). LOD = 3.3(Standard Error/Slope) and LOQ = 10*(Standard Error/Slope).*

**Brand**	**Brand A**			**Brand B**			**Brand C**		
**Buffer**	**PBS**	**Cit pH 6**	**Cit pH 4**	**PBS**	**Cit pH 6**	**Cit pH 4**	**PBS**	**Cit pH 6**	**Cit pH 4**
**Average % Diff.**	18.9	31.2	81.9	18.4	23.6	66.2	32.8	27.5	86.4
**RSD (%)**	25.7	20.2	44.5	21.5	25.8	18.5	36.6	16.6	6.6
**R^2**	0.9988	0.9987	0.8436	0.9993	0.9997	0.9985	0.9996	0.9986	0.9976
**LOD (ng/mL)**	70.1	86.3	72.6	1068.1	855.8.	3983.4	107.4	111.2	1177.3
**LOQ (ng/mL)**	212.4	261.4	220.0	3236.7	2593.4	12071.0	325.5	336.9	3567.5

Nanodrop analysis of branded Poly(A) samples (0.5–2.0 mg/mL) across different formulation buffers revealed significant discrepancies between theoretical and experimental concentrations, primarily due to salt content in lyophilised forms. Experimental concentrations differed by 18.3–32.8% in PBS and citrate pH 6 buffers, and up to 86.4% in citrate pH 4 **(Figure S 1, Table S 1)**, emphasising the impact of residual salts upon reconstitution. Despite strong linearity in calibration curves ($R^2 > 0.997$), Brand A in pH 4 citrate showed reduced linearity ($R^2 = 0.8436$), further indicating buffer-dependent variability. Lower absorbance at 260 nm in acidic buffers suggested conformational changes affecting adenine exposure and quantification accuracy. LODs and LOQs varied widely between brands and buffers, with up to an 11-fold difference **(Table S 1)**, reinforcing the importance of pre-checking Poly(A) concentrations via Nanodrop before downstream applications like nanoparticle encapsulation.

1.2 Dynamic Light Scattering

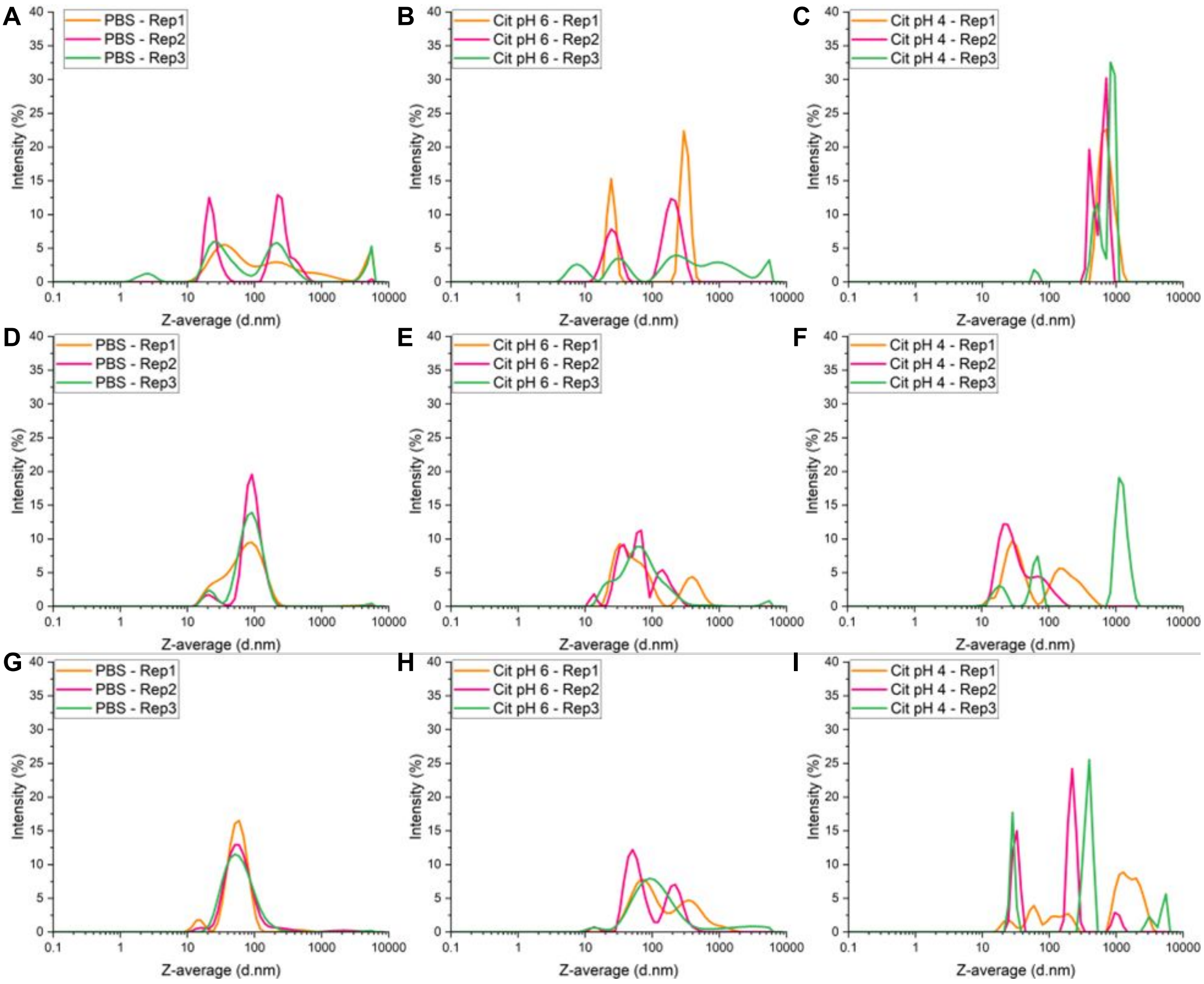

Figure S 2 - Intensity-based light scattering profiles of Poly(A) brand manufacturer drug substances in different formulation buffers with A) Brand A, B) Brand B and C) Brand C (n=3).

Table S 2 - DLS Z-average, polydispersity index, and corresponding three highest intensity peak sizes (n=3 ± SD).

Brand	Buffer	Z-Average (d.nm)	PDI	Peak 1 (nm)	Peak 2 (nm)	Peak 3 (nm)
Brand A	1× PBS pH 7.4	193.1 ± 44.2	0.337 ± 0.064	270	21	5560
	Citrate pH 6	186.8 ± 49.1	0.311 ± 0.039	295	24.4	5560
	Citrate pH 4	845.4 ± 13.0	0.248 ± 0.076	955	68.1	-
Brand B	1× PBS pH 7.4	66.4 ± 9.8	0.288 ± 0.076	91.1	21	-
	Citrate pH 6	54.5 ± 2.2	0.442 ± 0.085	58.8	37.8	396

	**Citrate pH 4**	128.0 ± 158.2	0.557 ± 0.264	24.4	1110	68.1
**Brand C**	**1× PBS pH 7.4**	49.5 ± 1.0	0.250 ± 0.010	58.8	15.7	-
	**Citrate pH 6**	82.9 ± 12.1	0.456 ± 0.039	58.8	190	13.5
	**Citrate pH 4**	291.5 ± 89.9	0.716 ± 0.219	28.2	220	396

DLS analysis of branded Poly(A) samples across formulation buffers revealed highly variable size and size distribution profiles, with broad polydispersity and inconsistent z-averages **(Figure S 2, Table S 2).** These differences reflect the limited light-scattering capability of RNA, particularly for smaller or highly polydisperse species. Brand-specific differences were observed, with Brand A showing the highest PDI in PBS and large aggregates in citrate pH 4 (z-average 955 nm). Brand B and C showed smaller average sizes and lower PDIs in PBS and citrate pH 6. Citrate pH 4 consistently led to multimodal distributions and irreversible sample gelation at analysed concentrations, regardless of manufacturer. Overall, DLS results were influenced by buffer conditions, RNA conformation, and gelation potential at low pH, highlighting the method's limitations for RNA characterisation due to weak scattering and high sample heterogeneity. Gelation potential was probed by measuring Poly(A) diffusion coefficients and calculating interaction parameter (K_D).

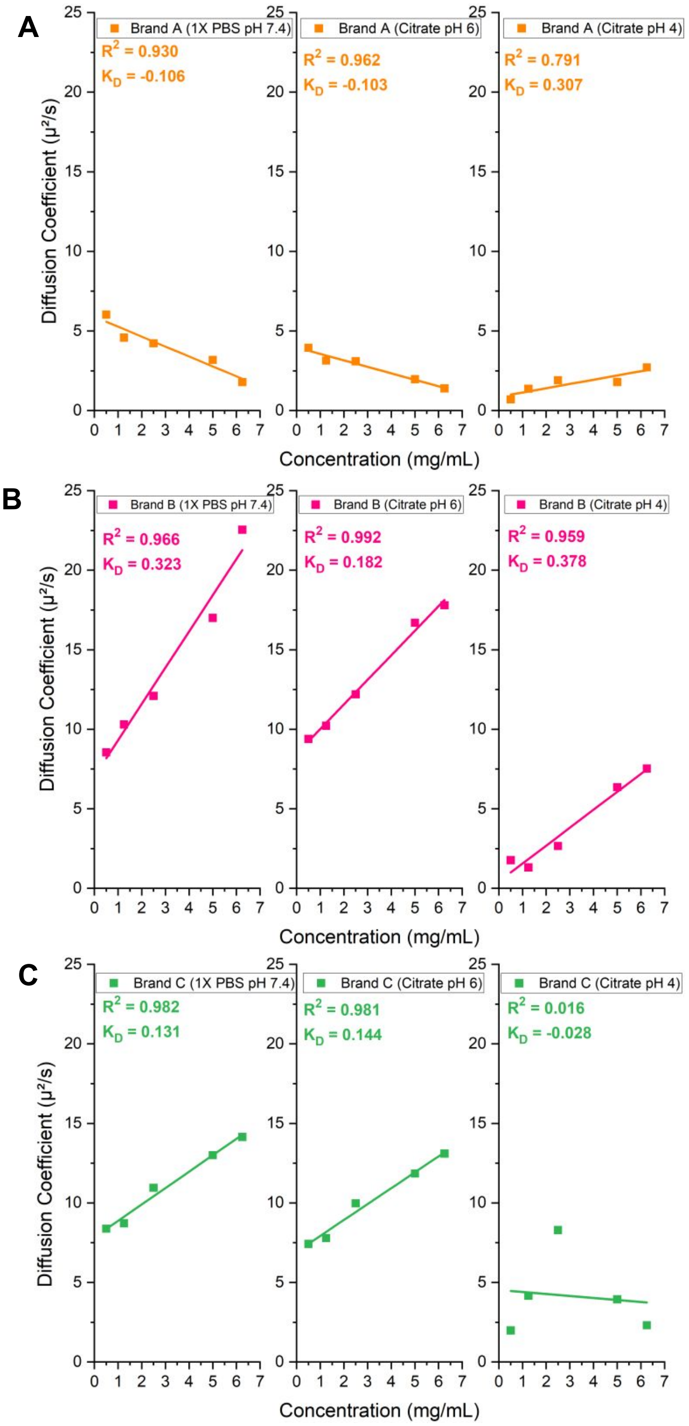

Figure S 3 - Concentration dependent diffusion coefficients over a series of Poly(A) concentrations in different formulation buffers (0.50-6.25 mg/mL) , A) Brand A, B) Brand B, and C) Brand C. K_D calculated from linear regression slope/intercept, n=2.

Calculated interaction parameter values (K_D) produced neither high repulsive values (positive K_D) nor high attractive values (negative K_D) **(Figure S 3),** indicating gelation noted was not detectable through diffusion coefficient measurements, however differences between sample buffers were noticed.

1.3 Capillary Gel Electrophoresis

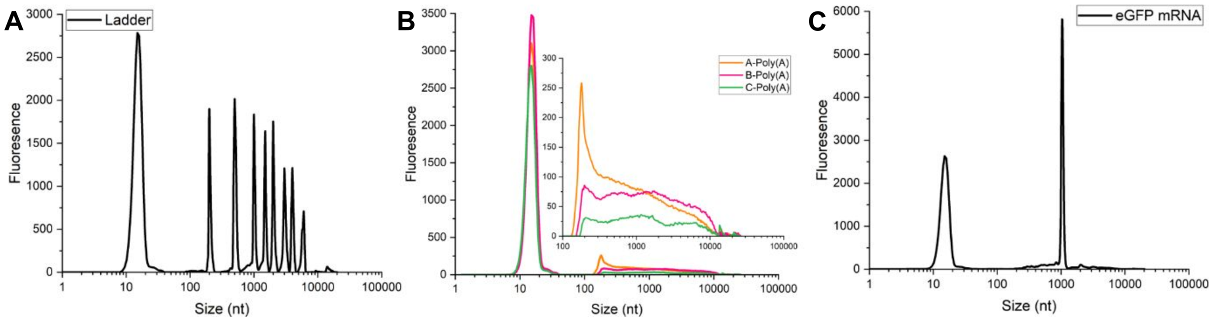

Figure S 4 - CGE evaluation of Poly(A) vendor specific chain length distribution profiles of A) RNA Ladder, B) A, B, C--Poly(A)s and C) eGFP mRNA control (n=3).

1.4 RiboGreen™ Assay Evaluation

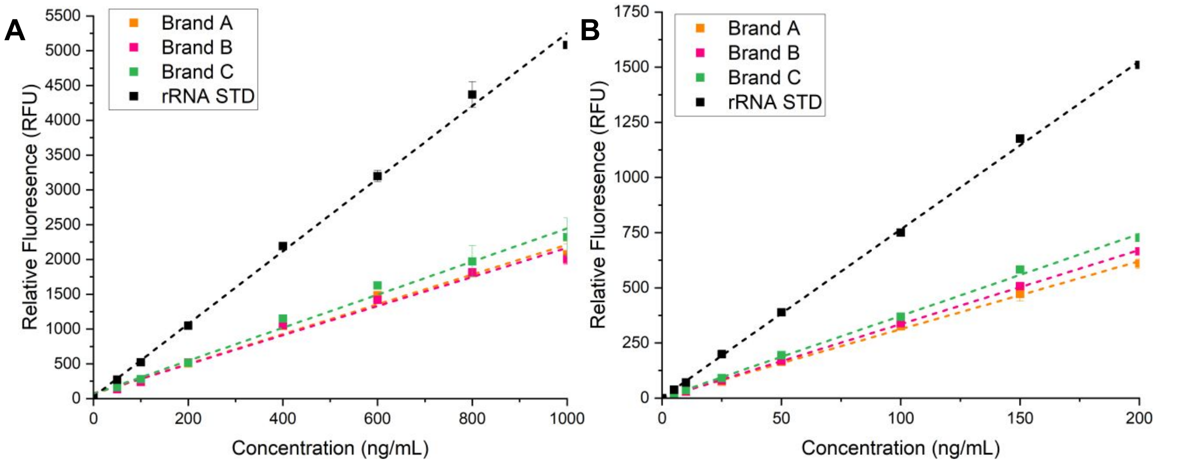

Figure S 5 - Calibration curves of Branded Poly(A) drug substances and rRNA Standard using RIboGreen™ Assay in A) Triton X-100 buffer and B) Tris-EDTA (TE) buffer, n=2 ± SD.

Table S 3 - Limit of Detection (LOD) and Limit of Quantification (LOQ) for Poly(A) in RiboGreen™ buffer conditions. Linear regression of calibration curves. LOD = 3.3(Standard Error/Slope) and LOQ = 10*(Standard Error/Slope), n=2.*

Brand	rRNA STD		Brand A		Brand B		Brand C	
Condition	Tri X	TE	Tri X	TE	Tri X	TE	Tri X	TE
R^2	0.998	0.999	0.987	0.999	0.984	0.999	0.990	0.998
LOD (ng/mL)	65.5	7.0	152.6	9.5	170.6	3.9	135.2	11.4
LOQ (ng/mL)	198.4	21.1	462.4	29.0	516.9	12.0	409.6	34.6

Table S 4 - RiboGreen™ concentration comparative differences between Poly(A) manufacturer drug substances, n=2, using one-way ANOVA Tukey test with samples analysed in Triton X-100 buffer. Ns = no statistical significance between means of relative fluorescence intensities per concentration level per Poly(A) manufacturer.

Conc. (ng/mL)	Brand A-B	Brand A-C	Brand B-C

**1000**	ns	ns	ns
**800**	ns	ns	ns
**600**	ns	$p < 0.05$	$p < 0.005$
**400**	ns	$p < 0.05$	ns
**200**	ns	ns	ns
**100**	ns	ns	ns
**50**	ns	ns	ns

Table S 5 - RiboGreen™ concentration comparative differences between Poly(A) manufacturer drug substances, n=2, using one-way ANOVA Tukey test with samples analysed in TE buffer. Ns = no statistical significance between means of relative fluorescence intensities per concentration level per Poly(A) manufacturer.

Conc. (ng/mL)	Brand A-B	Brand A-C	Brand B-C
200	ns	$p < 0.05$	ns
150	ns	$p < 0.05$	ns
100	ns	$p < 0.05$	$p < 0.05$
50	ns	$p < 0.05$	$p < 0.05$
25	ns	ns	ns
12.5	ns	ns	ns
5	ns	ns	ns

Table S 6 - NTA evaluation of Branded Poly(A) LNP formulations, (n=3 ± SD).

LNP	NTA						
	Mean (nm)	Mode (nm)	D10 (nm)	D50 (nm)	D90 (nm)	Span	Est. Conc ($x10^{11}$ particles/mL)
Brand A	68.3 ± 1.0	62.0 ± 0.9	46.8 ± 0.4	64.4 ± 1.3	90.4 ± 3.0	0.68 ± 0.03	2.35 ± 0.60
Brand B	66.8 ± 1.6	60.7 ± 1.6	45.5 ± 1.3	63.3 ± 1.7	90.2 ± 3.1	0.71 ± 0.04	3.04 ± 0.84
Brand C	68.8 ± 3.2	61.3 ± 3.4	46.2 ± 1.9	64.4 ± 3.1	92.9 ± 4.5	0.73 ± 0.01	2.54 ± 0.10

Table S 7 - FI-AF4-MD evaluation of Branded Poly(A) LNP formulations and associated cumulative distribution values for radius of gyration, hydrodynamic radius and shape factor. Span values and shape factor distributional standard deviations not shown as rounding would produce standard deviation of ± 0.0 due to reproducible replicates. (n=3 ± SD).

LNP	FI-AF4-MD													
	Et (min)	Rec (%)	R_G Mode	R_G 10 (nm)	R_G 50 (nm)	R_G 90 (nm)	R_G Span	R_H 10 (nm)	R_H 50 (nm)	R_H 90 (nm)	R_H Span	SF 10	SF 50	SF 90	SF Span
Brand A	22.3	88.8 ± 1.3	24.7 ± 0.1	23.1 ± 0.4	24.8 ± 0.2	27.1 ± 0.4	0.159	27.3 ± 0.8	29.9 ± 0.8	32.3 ± 0.9	0.167	0.824	0.838	0.856	0.038
Brand B	21.9	91.9 ± 7.1	24.2 ± 0.2	22.4 ± 0.5	24.1 ± 0.3	26.1 ± 0.2	0.152	26.9 ± 1.1	29.5 ± 0.8	32.0 ± 0.5	0.174	0.807	0.822	0.839	0.040
Brand C	22.3	92.6 ± 8.5	24.6 ± 0.3	22.8 ± 0.2	24.7 ± 0.2	27.4 ± 0.4	0.189	27.3 ± 1.0	30.1 ± 0.9	33.3 ± 0.6	0.200	0.809	0.826	0.847	0.046

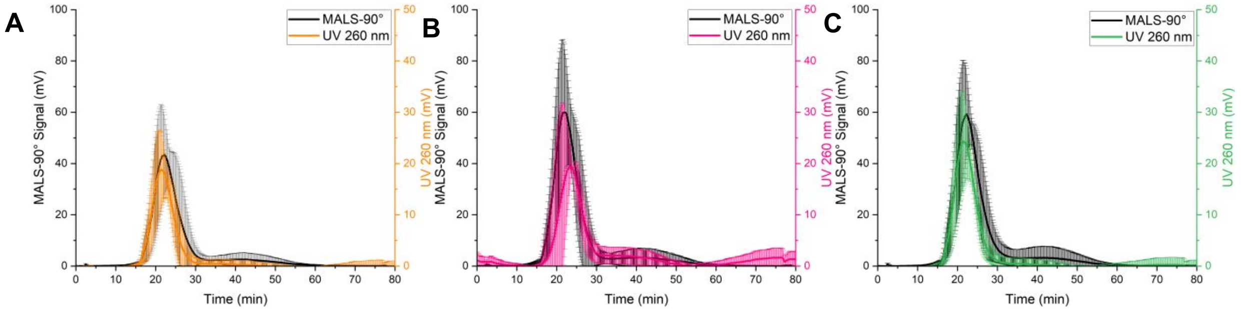

Figure S 6 -- FI-AF4-MD of Poly(A) LNP formulations. MALS-90° and UV (260 nm) elution profiles or A) A-LNPs, B) B-LNPs, and C) C-LNPs. (n=3 ± SD).

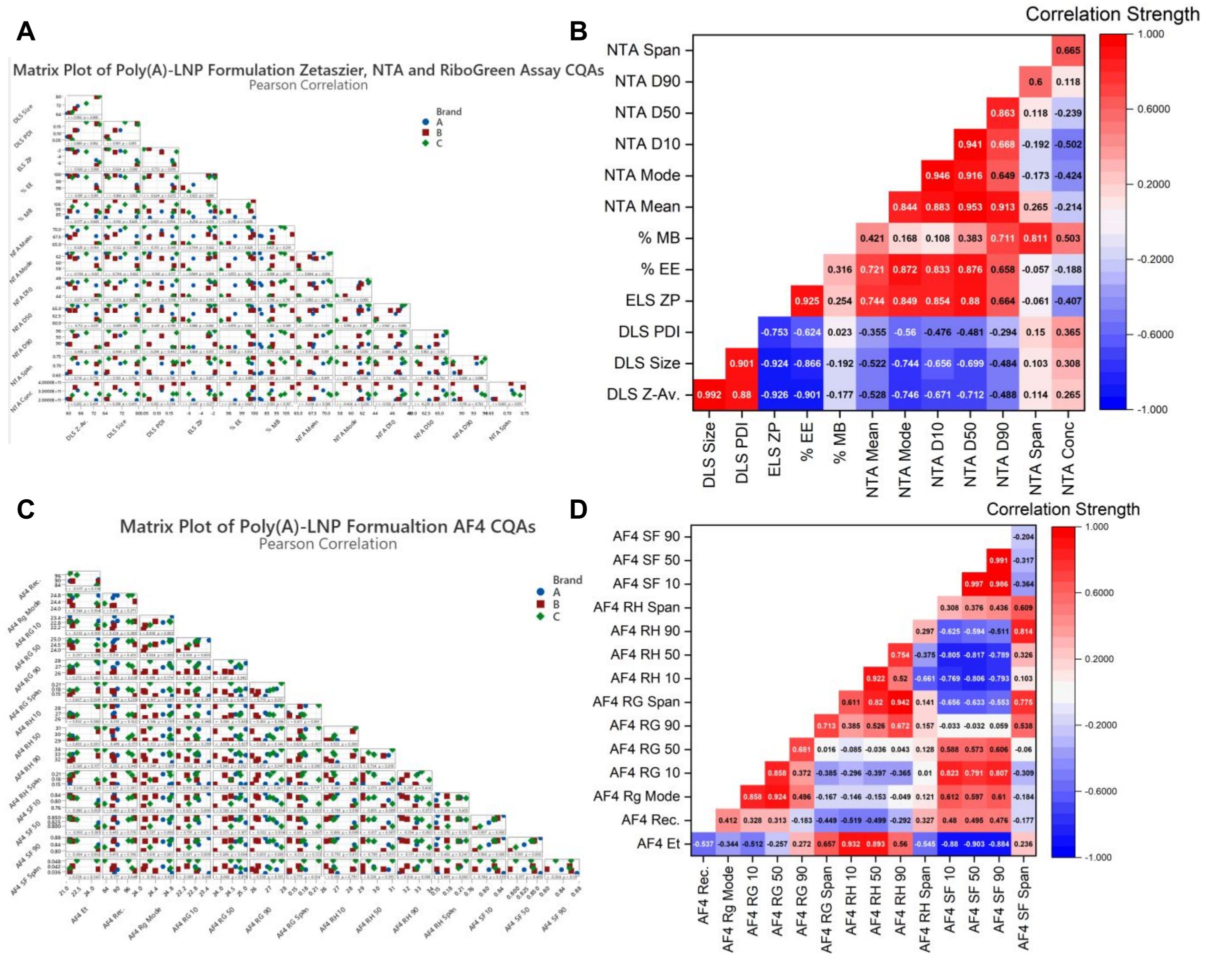

Figure S 7 – Correlation matrix and heatmap correlation strength plots of evaluated Branded Poly(A)-LNP drug product CQAs split by technique pipelines. A) Matrix plot of Zetasizer, RiboGreen™, and NTA CQAs, B) correlation strengths of Zetasizer, RiboGreen™, and NTA CQAs. C) Matrix plot of FI-AF4-MD CQA outputs and D) correlation strengths of FI-AF4-MD CQA outputs.
